# Cyclophilin–CD147 interaction enables SARS-CoV-2 infection of human monocytes and their activation via Toll-like receptors 7 and 8

**DOI:** 10.3389/fimmu.2025.1460089

**Published:** 2025-02-03

**Authors:** Gabor Tajti, Laura Gebetsberger, Gregor Pamlitschka, Katharina Aigner-Radakovics, Judith Leitner, Peter Steinberger, Hannes Stockinger, Anna Ohradanova-Repic

**Affiliations:** ^1^ Medical University of Vienna, Center for Pathophysiology, Infectiology and Immunology, Institute for Hygiene and Applied Immunology, Vienna, Austria; ^2^ Medical University of Vienna, Center for Pathophysiology, Infectiology and Immunology, Institute of Immunology, Vienna, Austria

**Keywords:** SARS-CoV-2, CD147, cyclophilin, Toll-like receptor, monocyte, antiviral innate immunity

## Abstract

Monocytes and macrophages, as important constituents of the innate immune system, are equipped with multiple Toll-like-receptors (TLRs) to recognize invading pathogens, such as SARS-CoV-2, and mount an antiviral response. Nevertheless, their uncontrolled activation can lead to hyperinflammation seen in severe COVID-19. Surprisingly, we observed that recombinant SARS-CoV-2 Spike (S) and Nucleocapsid (N) proteins triggered only a weak proinflammatory response in human peripheral blood monocytes. By employing THP-1 and Jurkat NF-κB::eGFP reporter cell lines expressing specific TLRs, various TLR ligands and blocking antibodies, we determined that surface TLRs, including TLR2/1, TLR2/6 and TLR4 do not play a major role in SARS-CoV-2 sensing. However, monocytes are potently activated by the replication-competent SARS-CoV-2, and the response correlates with the viral uptake that is observed only in monocytes, but not in lymphocytes. We show that monocyte activation involves two distinct steps. Firstly, SARS-CoV-2 infects monocytes in a process independent of the S protein and the prime SARS-CoV-2 receptor angiotensin-converting enzyme 2. Instead, the alternative SARS-CoV-2 receptor CD147, which is highly expressed on monocytes, recognizes its well-known interaction partners cyclophilins A and B that are incorporated into SARS-CoV-2 virions. Secondly, upon viral uptake via the cyclophilin-CD147 interaction, that can be inhibited by specific CD147 blocking antibodies or competition with recombinant human cyclophilin A and B, SARS-CoV-2 RNA is recognized by TLR7/8 in endosomes, leading to upregulation of tumor necrosis factor (TNF), interleukin (IL)-1β and IL-6, comprising the core hyperinflammatory signature. Taken together, our data reveal a novel mechanism how human monocytes sense SARS-CoV-2 and suggest that targeting the cyclophilin-CD147 axis might be beneficial to alleviate overt myeloid-driven inflammation triggered by SARS-CoV-2 infection.

## 1 Introduction

Severe acute respiratory syndrome coronavirus 2 (SARS-CoV-2), the causative agent of Coronavirus disease 2019 (COVID-19) is an enveloped, positive-sense single-stranded (ss)RNA virus belonging to the *Betacoronavirus* genus in the *Coronaviridae* family. SARS-CoV-2 causes relatively mild upper and lower respiratory tract infections, but some patients develop severe disease with acute respiratory distress syndrome (ARDS), systemic inflammation, tissue damage, and thromboembolic complications that can have fatal outcomes (1, 2). SARS-CoV-2 virions contain four structural proteins: the Spike (S), Membrane (M), and Envelope (E) proteins are embedded in the lipid envelope, while the Nucleocapsid (N) protein is complexed with genomic RNA in the virion core (1, 2). The trimeric S glycoprotein acts as the main determinant of SARS-CoV-2 tropism and via its receptor-binding domain (RBD) binds to the cardinal entry receptor angiotensin-converting enzyme 2 (ACE2) on the surface of susceptible cells, followed by proteolytic priming by the cell surface protease TMPRSS2, or alternatively, endosomal cathepsin L (3, 4). The S protein also engages other host cell receptors, such as heparan sulfate proteoglycans (HSPGs) (5–7), neuropilin-1 (NRP-1, CD304) (8, 9), L-SIGN (CLEC4M) (10) or heat shock protein GRP78 (11), all of which aid ACE2-mediated cell entry, thus fulfilling a coreceptor function. Additionally, S protein via its RBD or N-terminal domain has been shown to interact with several alternative receptors to mediate ACE2-independent SARS-CoV-2 entry (12), including CD147 (also known as basigin or EMMPRIN) (13), AXL (14), TMEM106B (15), RAGE (16), KREMEN1 (17), C-type lectin receptors such as CD209 (DC-SIGN), L-SIGN (CLEC4M), LSECtin (CLEC4G), CD301 (CLEC10A) and ASGR1 (10, 17–19), but also GRP78 (20). Lastly, the low affinity Fc receptor CD16 has been reported to enable infection of monocytes by binding antibody-opsonized SARS-CoV-2 (21).

The steps in viral entry and subsequent SARS-CoV-2 replication in the cytoplasm of infected cells present several opportunities for the innate immune system to sense viral components (1, 2). SARS-CoV-2 S, E and M proteins are exposed to host surface sensors during the attachment step, and host intracellular (cytoplasmic and endosomal) sensors can detect viral proteins and RNA upon entry, leading to activation of inflammatory signaling pathways and cell death (2, 22). Innate immune cells, especially monocytes and macrophages, are equipped with a wide range of pattern recognition receptors (PRRs), of which Toll-like receptors (TLRs) are the most prominent sensors of pathogen- or damage-associated molecular patterns (PAMPs or DAMPs, respectively) (2, 22, 23). TLRs generally transduce signals via two key adaptor molecules, MyD88 and TRIF, to activate downstream signaling cascades, which involve nuclear factor (NF)-κB and mitogen-activated protein kinases (MAPKs), leading to the induction of inflammatory cytokines, such as tumor necrosis factor (TNF) and interleukin-6 (IL-6), or interferon (IFN) regulatory factors (IRFs) that stimulate transcription of type I IFNs (2, 22, 23). In myeloid cells, type I IFNs also promote the expression of IL-15, a known antiviral cytokine (24), which has the capacity to counteract a concurrent T helper type 2 cell-dependent cytokine storm (25). Furthermore, transient upregulation of IL-15 in response to SARS-CoV-2 infection or SARS-CoV-2 vaccination is indicative of an efficient anti-viral immune response and viral clearance following infection with SARS-CoV-2 (26).

Several cell surface TLRs have been described to sense SARS-CoV-2 (2, 23, 27). TLR2, as a heterodimer with either TLR1 or TLR6, that normally recognizes e.g., triacylated (Pam3CSK4) or diacylated (FSL-1) lipoproteins, respectively (22), has been shown to act as a sensor of the SARS-CoV-2 S (28), or N (29) or E protein (30, 31) in macrophages or endothelial cells. These studies, however, yielded rather contradictory results since they pinpointed the TLR2 activation to a single SARS-CoV-2 structural protein and ruled out the others. Moreover, TLR4, a well-known sensor of lipopolysaccharide (LPS) of Gram-negative bacteria, was postulated to sense the SARS-CoV-2 S protein (32–35), while others excluded their direct interaction (36). Lastly, TLR1 through interaction with SARS-CoV-2 E and M proteins has been proposed to play a role in viral entry and the subsequent proinflammatory response in human and mouse myeloid cells (37).

SARS-CoV-2 may also activate endosomal single- or double-stranded RNA sensors TLR7, TLR8 (both sensing ssRNA) and TLR3 (sensing dsRNA) (22, 27, 38). Inborn errors in TLR3 and TLR7 have been linked to severe COVID-19 disease (38–40), suggesting that these TLRs may have a protective role during SARS-CoV-2 infection. Surprisingly, only one study so far has validated TLR3 sensing of SARS-CoV-2, showing that TLR3 activation upon infection of multicellular lung spheroids leads to type I IFN and cytokine production via the IRF3 signaling pathway (41). Sensing of SARS-CoV-2 RNA fragments via TLR7 or TLR8 has been predicted and experimentally validated in several studies (41–44). Additionally, SARS-CoV-2 has been shown to directly activate plasmacytoid dendritic cells (pDCs) via TLR7 and TLR2. While TLR7-MyD88 signaling was crucial for the production of antiviral IFNs, stimulation of TLR2 rather led to the inflammatory IL-6 response (9). However, an uncontrolled proinflammatory response of myeloid cells, especially monocytes and macrophages, upon SARS-CoV-2 sensing might lead to life-threatening systemic hyperinflammation or even cytokine storm (23, 45). By employing single-cell RNA sequencing of inflamed lungs of COVID-19 patients, SARS-CoV-2 RNA has been consistently detected in monocytes and macrophages (46–48), but subsequent *in vitro* studies yielded inconclusive results regarding whether these cells are directly infected or only able to bind and sense SARS-CoV-2 or its components (16, 19, 20, 49–51).

Motivated by these findings we set out to ascertain how human blood monocytes respond to infection with replication-competent SARS-CoV-2. We show that human monocytes can sense SARS-CoV-2 and subsequently mount a substantial proinflammatory response. SARS-CoV-2 sensing is not mediated via engagement of surface TLRs, but rather depends on infection via an ACE2-independent mechanism involving the alternative receptor CD147 and triggering endosomal ssRNA-sensing TLR7/8.

## 2 Materials and methods

### 2.1 Reagents and antibodies

Detailed description of reagents and antibodies used can be found in **Supplementary Table S1** in the **Supplementary Material**.

### 2.2 Primary cells and cell lines

Leukocyte reduction system chambers containing human blood cells of healthy, COVID-19-vaccinated donors were obtained from the Medical University of Vienna, Department of Transfusion Medicine and Cell Therapy, Vienna, Austria. Usage for research purposes was carried out in accordance with the Declaration of Helsinki and approved by the Ethics Committee of the Medical University of Vienna (2001/2018, 1238/2024). Peripheral blood mononuclear cells (PBMCs) were subsequently isolated via density gradient centrifugation using Lymphoprep and cryopreserved in 2-5 × 10^7^ cell aliquots. For experiments, PBMCs were thawed and seeded at a density of 1 × 10^6^ cells/ml in RPMI 1640 medium, supplemented with 2 mM L-glutamine, 100 U/ml penicillin, 100 μg/ml streptomycin, 10% FCS and 5 mM HEPES, hereafter referred to as complete RPMI. Cells were kept in culture for 24 h before treatment or infection.

Primary monocytes were sorted from the defrosted PBMCs via magnetic-activated cell sorting (MACS) using CD14 microbeads (Miltenyi Biotec) according to the manufacturer’s protocol and cultured in complete RPMI supplemented with 5 ng/ml M-CSF at a density of 1 × 10^6^ cells/ml for 24 h before treatment or infection. Human CD4^+^ T cells were isolated from CD14-depleted PBMC fraction by negative selection using CD8, CD16, CD19, CD20 and CD56 mAbs (a kind gift of Prof. Vaclav Horejsi) and anti-mouse IgG microbeads (Miltenyi Biotec), as done previously (52).

Human lymphocytic Jurkat NF-κB::eGFP and human monocytic THP-1 NF-κB::eGFP reporter cell lines were generated as described elsewhere (53–55). Jurkat reporter cell lines employed in this study expressed either TLR2 and TLR1 (Jurkat–TLR2/1), TLR2 and TLR6 (Jurkat–TLR2/6), TLR4, MD2 and CD14 (Jurkat–TLR4/MD2/CD14) or no TLR (Jurkat–no TLR), in which the endogenously expressed TLR5 was inactivated using CRISPR/Cas9 (55). THP-1 reporter cells were not engineered with respect to TLR expression, thereby the endogenous set of TLRs were present. Reporter cell lines were cultured in complete RPMI without HEPES, at a density of up to 1 × 10^6^ cells/ml.

Human colon adenocarcinoma-derived Caco-2 cells (ATCC HTB-37), human lung adenocarcinoma-derived Calu-3 cells (ATCC HTB-55), Lenti-X 293T cells (Takara Bio) and African green monkey kidney-derived Vero cells (ATCC CCL-81) were cultured in DMEM, supplemented with 100 U/ml penicillin, 100 μg/ml streptomycin, and 10% FCS, hereafter referred to as DMEM/10% FCS. Serum concentration in the medium was reduced to 2% for infections with SARS-CoV-2 (DMEM/2% FCS).

### 2.3 SARS-CoV-2 infection, virus stock production and quantification

SARS-CoV-2 isolate BetaCoV/Munich/BavPat1/2020, kindly provided by Prof. Christian Drosten, Charité, Berlin (56) and distributed by the European Virology Archive (Ref-SKU: 026V-03883), was propagated in Caco-2 cells that were shown to produce high virus titers without overt contamination of the viral stocks with inflammatory cytokines (51). Accordingly, to produce SARS-CoV-2 stocks, Caco-2 cells were seeded in DMEM/10% FCS in T-75 cm^2^ tissue culture flasks to reach 80% confluency 24 h later, when medium was exchanged to DMEM/2% FCS and cells were infected with SARS-CoV-2 at multiplicity of infection (MOI) of 0.05. Virus-containing cell culture supernatants were harvested after 72 h, cleared by centrifugation (2000 g, 10 min, 4°C), aliquoted, and frozen at -80°C. In parallel, we also generated mock cell culture supernatants of Caco-2 cells using the same procedure but omitting SARS-CoV-2 inoculum.

Virus titers were determined via the 50% tissue culture infectious dose (TCID_50_) assay using Vero cells as described before (57). The same assay was used to quantify SARS-CoV-2 in cell culture supernatants harvested from infected monocytes and Caco-2 cells.

For experiments, primary monocytes, Caco-2 cells, or reporter cell lines were seeded in 24-well plates, rested for 24 h, infected with SARS-CoV-2 produced by Caco-2 cells at MOI 2, and harvested after 24 h unless otherwise stated. Cells were usually incubated with SARS-CoV-2 or equal volume of mock supernatant during the entire incubation time, but in virus entry-blocking experiments, excess virus was washed away after 1 h incubation at 37°C. TLRs were inhibited by an anti-TLR2 monoclonal antibody (mAb) or treated with an isotype control mAb (both Miltenyi Biotec, and used at final 10 µg/ml), or by TLR7/8 inhibitor enpatoran (MedChemExpress; final 1 µM) 1 h before SARS-CoV-2 infection. Similarly, SARS-CoV-2 entry was blocked by applying soluble SARS-CoV-2 Spike-RBD (sRBD, BioLegend; 5 µg/ml), heparin (Sigma-Aldrich, 250 U/ml), CD147 mAb (a kind gift of Prof. Vaclav Horejsi), CD301 mAb (BioLegend), or an isotype control mAb, each 20 µg/ml), or recombinant human cyclophilin A and cyclophilin B (both MedChemExpress, and used at final 10 µg/ml) for 1 h before infection, and in case the excess virus was washed away after 1 h of incubation, the treatment was re-applied. All experiments involving replication-competent SARS-CoV-2 were performed in the Biosafety Level 3 (BSL-3) facilities of the Medical University of Vienna.

### 2.4 Dissection of the TLR-dependent pathways

TLR agonists, Pam3CSK4 for TLR2/1, FSL-1 for TLR2/6, ultrapure LPS for TLR4/MD2/CD14 and the dual TLR7 and TLR8 synthetic agonist resiquimod (R848) were added to monocytes or NF-κB::eGFP reporter cell lines for 24 h. While monocytes were treated with a single concentration (see figure legends), a range of concentrations was used for the reporter cell lines. If treatments were combined with the TLR2 blocking (or isotype control) mAb used at 10 µg/ml, cells were pre-incubated with mAbs for 30 min before applying the agonists. Recombinant SARS-CoV-2 S or N protein (both at 1 µg/ml) were also added for 24 h. Cells were then harvested and analyzed by flow cytometry or RT-qPCR.

### 2.5 Generation of the novel TLR7 and TLR8 reporter cell lines

For cloning of the full-length TLR7 and TLR8, complementary DNA (cDNA) was generated from human monocyte-derived macrophages, differentiated with M-CSF and activated with LPS+IFN-γ, that express both ssRNA-sensing receptors at high levels (52). An open reading frame (ORF) of TLR7 was amplified by PCR using primers flTLR7f (5´-ACCAGACCTCTACATTCCATTTTG-3´) and flTLR7r (5´-AGGGCTAGACCGTTTCCTTG-3´) and a Phusion high-fidelity DNA polymerase, gel purified using Monarch DNA Gel Extraction Kit and ligated into EcoRV-linearized pBluescript KS(-) vector (Stratagene). The cloned sequence was verified by Sanger sequencing and recloned in the correct orientation into the retroviral vector pBMN-Z via blunt-ended HindIII site and NotI site. Similarly, the TLR8 ORF was amplified using primers flTLR8f (5´-TTGAAAGGGAGAATGAAGGAGTC-3´) and flTLR8r (5´- TCATTCCTTTGCATCTTTATTATGG-3´) and recloned into pBMN-Z opened with BamHI and blunt-ended NotI.

TLR7 and TLR8 were delivered into THP-1 NF-κB::eGFP reporter cells by retroviral transduction as previously described (58). Briefly, Lenti-X 293T cells (Takara Bio) were transfected with the pBMN-Z-TLR7 or pBMN-Z-TLR8 retroviral plasmids and the packaging plasmids pMD_OGP and pMD2.G using TurboFect (Thermo Fisher Scientific). Viral supernatants were harvested after 48 h, filtered, and used for the overnight transduction in the presence of 5 µg/ml polybrene. After a week, the THP-1 NF-κB::eGFP reporter cell cultures, including mock-transduced cells used as a negative control, were stimulated with 0.3 µg/ml resiquimod overnight, and transduced cells, expressing the reporter gene eGFP at high levels, were sorted using a SH800 cell sorter (Sony Biotechnology). The resulting cultures were expanded and probed for the presence of transgenes using qRT-PCR and for the sensitivity to resiquimod.

### 2.6 SARS-CoV-2 RNA cloning, *in vitro* transcription and transfection

SARS-CoV-2 genomic RNA isolated from the supernatant of infected Calu-3 cells was transcribed into cDNA using the High-Capacity cDNA Reverse Transcription Kit (Applied Biosystems) and a NSP4-9r primer (5´-**TTA**TTGTAGACGTACTGTGGCAGCTA-3´, incorporating a stop codon in bold). Afterwards, cDNA spanning nonstructural proteins (NSP) 4 to NSP9 (genome position 8495-12970) was amplified with a Phusion polymerase and primers NSP4-9f (5´-**CAACATG**GGTGGTAAAATTGTTAATAATTGGTTG-3´, incorporating a Kozak consensus sequence and a start codon, in bold) and NSP4-9r. The resulting 4.5 kb-long PCR product was gel purified, ligated into EcoRV-linearized pBluescript KS(-) vector and verified by Sanger sequencing. The clone with a forward orientation of the insert in respect to the T7 promoter was linearized with XhoI, gel purified and 1 µg of the linearized DNA was used as a template for *in vitro* transcription using the HiScribe T7 Quick High Yield RNA Synthesis Kit (New England Biolabs). The template was afterwards removed using DNAse I. One µg of SARS-CoV-2 NSP4-9r RNA was used to transfect 6 × 10^5^ primary monocytes (or 8 × 10^5^ THP-1 NF-κB::eGFP reporter cells) with Lipofectamine 3000 (Thermo Fisher Scientific), following the protocol of the manufacturer.

### 2.7 Flow cytometry

SARS-CoV-2-infected PBMCs were harvested, blocked with 4.8 mg/ml human IgG (3% Beriglobin P, CSL Behring) and stained for the lineage surface markers (CD3, CD4, CD8, CD14, CD16, CD19, CD56) using mAbs (BioLegend or BD Biosciences) specified in **Supplementary Table S1**, as done previously (52). Afterwards, cells were fixed with 5% methanol-free formaldehyde for 20 min at room temperature (RT), permeabilized with 0.1% saponin in PBS for 15 min at RT, and from this point, maintained in intracellular staining buffer (PBS containing 5% FCS, 0.1% saponin and 0.02% NaN_3_). Cells were re-blocked with 1.6 mg/ml human IgG (1% Beriglobin P) and stained using rabbit anti-SARS-CoV-2 N protein mAb (Sino Biological), followed by Alexa Fluor (AF) 647-conjugated goat anti-rabbit IgG (H+L) secondary antibody (Invitrogen). Reporter cells were only washed with PBS, fixed (if infected), washed again and directly analyzed. Data were acquired using a Cytek Aurora spectral flow cytometer equipped with SpectroFlo software (Cytek Biosciences), or alternatively, using an LSRFortessa flow cytometer (BD Biosciences), and analyzed using FlowJo 10 software (BD Biosciences). Signals were expressed as geometric mean of fluorescence intensity (gMFI), and in case of the reporter cells, normalized to the gMFI of untreated samples.

### 2.8 Microscopy

SARS-CoV-2-infected monocytes were harvested after 24 h, stained with the Brilliant Violet 421-conjugated CD14 mAb (BioLegend), washed, fixed, permeabilized and stained with AF488-conjugated anti-LAMP-1 mouse mAb (BioLegend) and with the chimeric anti-SARS-CoV-2 N protein mAb (Absolute Antibody), followed by AF647-conjugated goat anti-rabbit IgG (H+L) secondary antibody (Invitrogen). Alternatively, infected monocytes and Caco-2 cells (grown in 8-well Lab-Tek chamber slides (NUNC)), were stained via indirect immunofluorescence for SARS-CoV-2 N protein as stated above, or for dsRNA using anti-dsRNA mAb (J2, Jena Bioscience) and AF647-conjugated goat anti-mouse IgG (H+L) secondary Ab (Invitrogen). Cell nuclei were stained using DAPI. After staining procedure, monocytes were cytospinned and mounted onto glass slides using CC/Mount while Caco-2 cells were only mounted onto chamber slides. Samples were imaged via fluorescence microscopy using an Eclipse Ti-E inverted microscope system (Nikon) with the equipment described elsewhere (59). Images were further processed using the Fiji software (60).

### 2.9 RNA isolation and gene expression analysis

Total RNA was extracted from cells using commercially available total RNA extraction kits (see **Supplementary Table S1**) according to the manufacturer’s instructions with minor modifications to comply with the biosafety requirements. After extraction, a standardized amount of RNA was reverse-transcribed to cDNA using the M-MuLV Reverse Transcriptase kit (New England Biolabs). Gene expression and SARS-CoV-2 N protein RNA within cells was measured via quantitative real-time PCR (qPCR) using Luna Universal qPCR Master Mix or Luna Universal Probe qPCR Master Mix (New England Biolabs), respectively, and primers [(52, 61) and this study] listed in **Supplementary Table S1**. qPCR data were recorded on the CFX96 Touch Real-Time PCR Detection System (Bio-Rad). For samples that failed to amplify, a CT value was set to 40. Data are shown as - ΔCT, i.e. log2 difference in expression compared to the *EEF1A1* housekeeping gene that was used for normalization.

### 2.10 ELISA

Recombinant His-tagged S protein (BioLegend) was coated overnight onto Pierce Nickel Coated Plates (Thermo Fisher Scientific) at 1 µg/ml at 4°C. Plates were subsequently washed 3 times with 0.05% Tween-20 in PBS (wash buffer). After blocking with 3% BSA in wash buffer for 1 h at RT, ACE2Fc (GenScript) or CD147Fc (Acro Biosystems) were added in duplicates at various concentrations and incubated for 1 h. After washing, the detection Ab (HRP-conjugated mouse anti-human IgG Fc mAb, at 1:10000 dilution) was added for 1 h, followed by washing 3 times, and the plates were developed with Substrate Reagent Pack (R&D Systems) for 20 min at RT and stopped with 2N HCl. Absorbance at 450 nm and 630 nm (for background correction) was measured with Mithras LB 940 microplate reader (Berthold Technologies). Values are plotted as OD450-OD630, and binding curves were generated with 4-parameter sigmoidal fit.

### 2.11 Purification of SARS-CoV-2 virions and Western blot analysis

Supernatants of Caco-2 cells infected with SARS-CoV-2 at MOI 0.05 for 72 h, as well as mock supernatants prepared in parallel were cleared from cell debris by centrifugation, and the majority was further concentrated using Amicon Ultra Centrifugal Filters with a 100 kDa cut-off (Merck Millipore), followed by purification with the Capto Core 700 resin (Cytiva), as described (62, 63). Afterwards, purified samples were lysed using 5x Laemmli buffer and boiled at 95°C for 10 min to inactivate infectious SARS-CoV-2. Lysates were subjected to reducing SDS-PAGE (10%) and Western blot analysis as we did previously (58) using antibodies specified in **Supplementary Table S1**, followed by chemiluminescent detection (ECL Prime Western Blot detection, Cytiva) and acquisition using LAS 4000 (Fujifilm). Images were analyzed using the Fiji software (60).

### 2.12 Statistical analysis

Statistical analysis and graphing were done using Prism 10 (GraphPad Software). Normality of data distribution was always tested with Shapiro-Wilk test. Statistical tests used for comparison are detailed in the corresponding figure legends.

## 3 Results

### 3.1 Recombinant SARS-CoV-2 S and N proteins trigger a weak proinflammatory response in primary human monocytes and the THP-1 and Jurkat NF-κB::eGFP reporter cell lines

**Figure 1:**
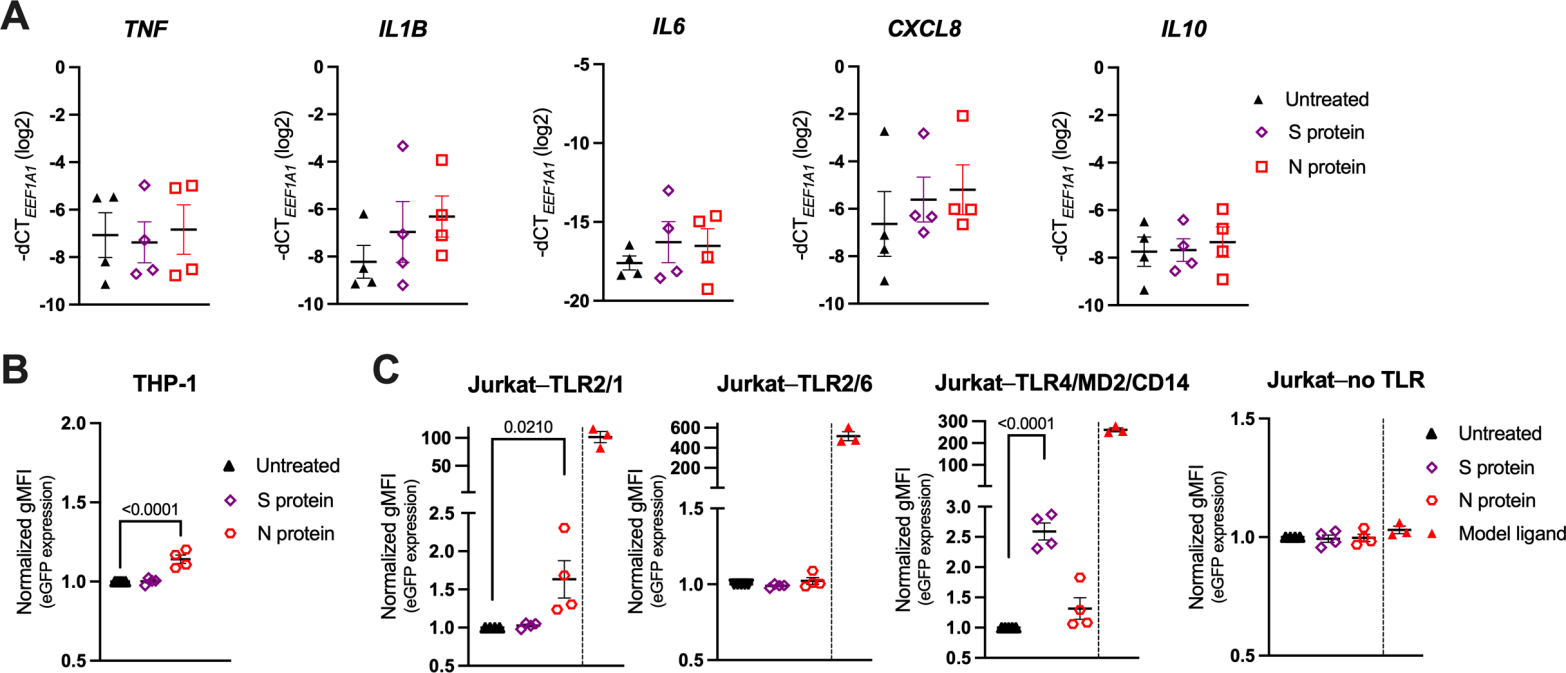
Recombinant SARS-CoV-2 Spike (S) and Nucleocapsid (N) proteins trigger only a weak proinflammatory response in primary human monocytes and the THP-1 and Jurkat NF-κB::eGFP reporter cells. **(A)** MACS-sorted human peripheral blood monocytes were treated with recombinant trimeric SARS-CoV-2 S or recombinant SARS-CoV-2 N protein (both at 1 μg/ml) or were left untreated. mRNA expression levels of the proinflammatory cytokines *TNF*, *IL1B*, *IL6*, *CXCL8* and *IL10* were determined by RT-qPCR after 24 h. Data are represented as mean ± standard error of the mean (SEM; n = 4 donors). **(B, C)** THP-1 NF-κB::eGFP reporter cells **(B)** or Jurkat NF-κB::eGFP reporter cells **(C)** engineered to express the indicated set of Toll-like receptors (TLRs) were treated with SARS-CoV-2 S or N protein (both at 1 μg/ml) or with the following model ligands: 100 ng/ml Pam3CSK4 (Jurkat–TLR2/1 reporter cells), 100 ng/ml FSL-1 (Jurkat–TLR2/6 reporter cells), 2 ng/ml LPS (Jurkat–TLR4/MD2/CD14 reporter cells), 100 ng/ml FSL-1 (Jurkat–no TLR reporter cells). Expression of the eGFP reporter gene was measured by flow cytometry after 24 h and depicted as fold-change of gMFI. Scatter plots show individual values and mean ± SEM (n = 4 independent experiments). S- and N-protein-treated samples in **(A–C)** were compared to the respective untreated cells using one-way ANOVA with Dunnett’s *post-hoc* test. Positive controls (separated by a dashed line) were excluded from the statistical analysis.

Since there is no consensus in the literature regarding the recognition of SARS-CoV-2 structural proteins by TLR2 or TLR4 (28–34, 36), we first assessed whether human peripheral blood monocytes, which express TLR2, TLR4 and the TLR4 coreceptor CD14 at high levels (20, 64, 65), reacted to the SARS-CoV-2 S or N protein. Accordingly, we incubated monocytes isolated from human PBMCs of healthy donors with either recombinant trimeric SARS-CoV-2 S protein, or recombinant SARS-CoV-2 N protein, both with declared negligible levels of endotoxin, for 24 h and determined their activation by measuring cytokine gene expression by reverse transcription-quantitative PCR (RT-qPCR). Both the S and N protein very mildly upregulated the proinflammatory genes *IL1B*, *IL6* and *CXCL8*, while *TNF* and the anti-inflammatory gene *IL10* remained unchanged (**Figure 1A**). To determine whether this mild activation occurred as a result of cell surface TLR engagement, we further treated the sensitive NF-κB::eGFP monocytic THP-1 reporter cell line, which expresses the endogenous TLR2/1, TLR2/6, TLR5, TLR7, TLR8 but not CD14 and/or TLR4 (53). Interestingly, THP-1 reporter cells reacted only minimally to the SARS-CoV-2 N protein, and not at all to the S protein (**Figure 1B**), while the NF-κB-driven upregulation of the reporter gene eGFP in response to TLR2/1 (Pam3CSK4), TLR2/6 (FSL-1) and TLR7/8 (resiquimod) model ligands, used as positive controls, was as expected (**Supplementary Figure S1A**, which can be found in **Supplementary Material**).

To further dissect a potential role of cell surface TLRs in SARS-CoV-2 sensing, we employed the highly sensitive Jurkat NF-κB::eGFP reporter cell lines that were engineered to express either TLR2/1, TLR2/6, or TLR4, together with the accessory molecule MD2 and the coreceptor CD14 (TLR4/MD2/CD14), as well as the NF-κB-driven eGFP reporter gene upon stimulation with the respective TLR ligand (**Figure 1C**; **Supplementary Figure S1B**) (55). Jurkat NF-κB::eGFP reporter cells without surface TLRs were used as a control (55). As shown in **Figure 1C**, treatment of these reporter lines with the SARS-CoV-2 S or N protein resulted in mild upregulation of eGFP in the Jurkat–TLR4/MD2/CD14 or Jurkat–TLR2/1 reporter cells, respectively, while much higher activation was observed upon treatment with the canonical TLR ligands (Pam3CSK4 for TLR2/1 and LPS for TLR4/MD2/CD14). Jurkat–TLR2/6 and Jurkat reporter cells without TLRs did not react to the SARS-CoV-2 proteins (**Figure 1C**). These data suggest that TLR2/1 and TLR4 might be engaged upon stimulation with the recombinant SARS-CoV-2 S and N proteins, however, the activation of the downstream NF-κB pathway is rather low.

### 3.2 Treatment of human monocytes with SARS-CoV-2 induces proinflammatory cytokine expression in a TLR2-independent manner

**Figure 2:**
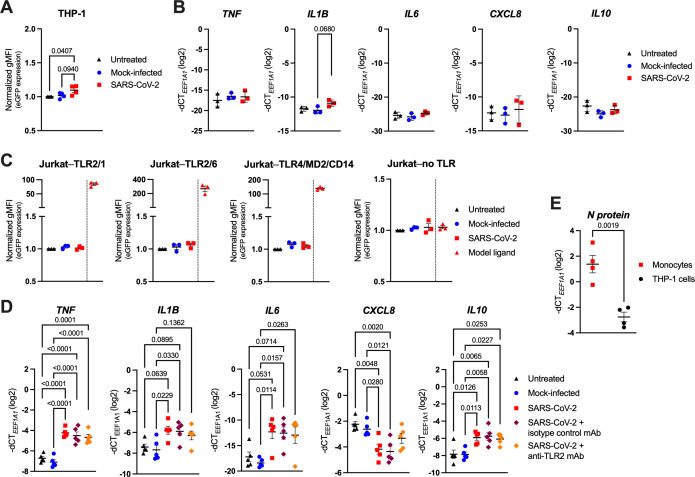
SARS-CoV-2 activates human monocytes, but not highly sensitive THP-1 or Jurkat NF-κB::eGFP reporter cells. **(A)** THP-1 NF-κB::eGFP reporter cells were infected with SARS-CoV-2 at MOI 2, or treated with the respective mock supernatant, or left untreated for 24 h. Afterwards, cells were fixed, washed, and eGFP expression was measured by flow cytometry. Data show normalized eGFP gMFI as mean ± SEM (n = 4 independent experiments). **(B)** RT-qPCR analysis of *TNF*, *IL1B*, *IL6, CXCL8* and *IL10* in THP-1 NF-κB::eGFP reporter cells infected with SARS-CoV-2 at MOI 2 for 24 h. Data are represented as mean ± SEM (n = 3 independent experiments). **(C)** Normalized eGFP gMFI of the indicated Jurkat NF-κB::eGFP reporter cells treated as the THP-1 NF-κB::eGFP reporter cells shown in **(A)**. Model ligands, as specified in **Figure 1C**, were used as positive controls. Data are represented as mean ± SEM (n = 3 independent experiments). **(D)** RT-qPCR analysis of *TNF*, *IL1B*, *IL6*, *CXCL8* and *IL10* in MACS-sorted human peripheral blood monocytes infected with SARS-CoV-2 at MOI 2, or treated with the respective mock supernatant, or left untreated for 24 h. Infection was also performed in the presence of the TLR2 blocking mAb (or an isotype control mAb; both at 10 μg/ml). Data are represented as mean ± SEM (n = 5 donors). **(E)** RT-qPCR analysis of SARS-CoV-2 N protein mRNA in human blood monocytes or THP-1 NF-κB::eGFP reporter cells infected as above. Data are represented as mean ± SEM (n = 4 independent experiments). Statistical significance in **(A-D)** was assessed by one-way ANOVA with Tukey’s *post-hoc* test (positive controls in **(C)** were excluded from the analysis), or by unpaired t-test in **(E)**.

As the stimulation with recombinant proteins was inconclusive, we next incubated THP-1 and Jurkat NF-κB::eGFP reporter cells as well as primary human monocytes with replication-competent SARS-CoV-2, followed by the quantification of the eGFP reporter gene and/or cytokine gene expression via RT-qPCR after 24 h (**Figures 2A–D**). Similarly to the viral proteins, SARS-CoV-2 only mildly increased eGFP and *IL1B* levels in the monocytic THP-1 reporter cells when compared to mock-infected (conditioned cell culture medium) or untreated (cell culture medium only) cells (**Figures 2A, B**), whereas the Jurkat reporters did not react at all (**Figure 2C**). In contrast, SARS-CoV-2 markedly activated primary human monocytes, reflected by the significant upregulation of *TNF*, *IL1B*, *IL6* and *IL10*, along with the downregulation of *CXCL8* (**Figure 2D**), suggesting a mechanism of activation at least partially independent of surface TLRs. Quantification of the SARS-CoV-2 N protein RNA levels by RT-qPCR further revealed a significantly higher intracellular viral load in primary monocytes compared to THP-1 cells (**Figure 2E**). This implies that the different capability of virus uptake may account for the observed differences in cytokine responses between the two cell types.

Due to the heavily proposed role of TLR2 in SARS-CoV-2 sensing (28–31), we also analyzed the cytokine expression of SARS-CoV-2-infected monocytes pretreated with a TLR2 blocking monoclonal antibody (mAb), or an isotype control mAb. Interestingly, we observed no significant changes in cell activation when TLR2 function was inhibited (**Figure 2D**), indicating that TLR2 may play only a minor part in SARS-CoV-2 sensing by human monocytes. We could not attribute this lack of efficacy to low TLR2 expression (**Supplementary Figure S2A**) nor low potency of the used blocking mAb, as stimulation of monocytes with TLR2/1 (Pam3CSK4) and TLR2/6 (FSL-1) model agonists effectively increased *IL1B, IL6* and *CXCL8* (though not *TNF* and *IL10*) expression, and TLR2 inhibition partially abrogated these changes (**Supplementary Figure S2B**). Moreover, the TLR2 blocking mAb used at the same concentration (10 µg/ml) completely abrogated the Pam3CSK4- or FSL-1-mediated activation of the TLR2/1- or TLR2/6-expressing Jurkat NF-κB::eGFP reporter cells (**Supplementary Figure S2C**), thereby further confirming its functionality.

Collectively, these data indicate that surface TLRs likely play an imperceptible role in SARS-CoV-2 sensing, but despite that, monocytes are able to both sense and respond to SARS-CoV-2, and the magnitude of their activation correlates with the amount of the virus bound or taken up by the cells.

### 3.3 Human monocytes are abortively infected by SARS-CoV-2

**Figure 3:**
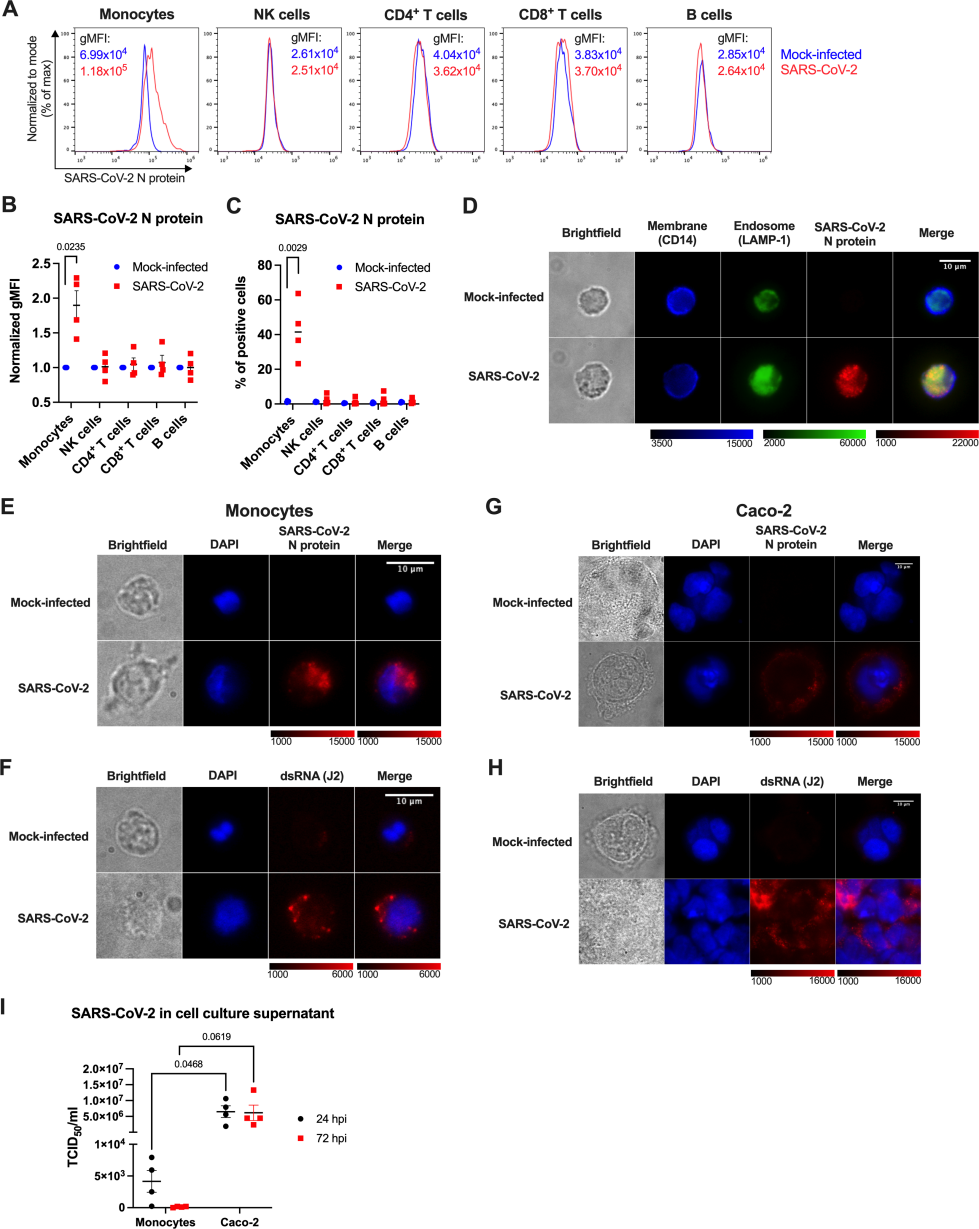
Human monocytes are abortively infected by SARS-CoV-2. **(A-C)** PBMCs of healthy donors were infected with SARS-CoV-2 at MOI 2 for 24 h, stained for cell surface lineage markers and intracellular SARS-CoV-2 N protein and analyzed by flow cytometry. Monocytes were identified as CD3^-^CD14^+^, NK cells as CD3^-^CD56^+^, B cells as CD3^-^CD19^+^ and CD3^+^ T cells as either CD4^+^ or CD8^+^ cells. N protein staining within the PBMC subsets of one representative experiment **(A)**, gMFI normalized to the mock-infected samples **(B)**, and percentage of the N-protein positive cells **(C)** in PBMCs of 4 donors are shown. **(D)** MACS-sorted monocytes infected with SARS-CoV-2 at MOI 2 for 24 h were stained for CD14 (a cell surface marker), LAMP-1 (an endosomal marker), and viral N protein and imaged by fluorescence microscopy. A representative image of 3 independent experiments is shown. **(E-I)** Monocytes **(E, F, I)** and Caco-2 cells **(G–I)** were mock-infected, or infected with SARS-CoV-2 at MOI 2 to visualize SARS-CoV-2 infection and replication **(E-H)** within cells at 24 hpi, or compare amounts of the produced SARS-CoV-2 at 24 and 72 hpi **(I)**. **(E, G)** Intracellular staining of SARS-CoV-2 N protein to visualize infection. **(F, H)** Intracellular staining of dsRNA using the J2 mAb to visualize SARS-CoV-2 replication. **(I)** Cell-free supernatants from infected monocytes and Caco-2 cells were harvested at 24 and 72 hpi, and immediately titrated using Vero cells. Virus titers were determined by the TCID_50_ assay. Data are represented as mean ± SEM (n = 4 independent experiments). Statistical significance was assessed using one sample t-test **(B)**, unpaired t-test **(C)**, or two-way ANOVA with Tukey’s *post-hoc* test **(I)**.

With the intention to ascertain whether only primary monocytes, or possibly other PBMC subsets can bind, or even be infected by SARS-CoV-2, we incubated PBMCs of healthy donors with replication-competent SARS-CoV-2 and analyzed cell surface lineage markers and the intracellular SARS-CoV-2 N protein by flow cytometry 24 h post infection (24 hpi). Monocytes, discriminated by CD14 staining, were identified as the only infected cell type, as shown by representative flow cytometry histograms (**Figure 3A**) and quantification based on normalized gMFI (**Figure 3B**). Depending on the donor, 23-64% monocytes scored positively (**Figure 3C**), and there was no quantitative difference in the staining intensity between classical (CD14^high^) and nonclassical (CD14^low^) monocytes (data not shown). Other PBMC subsets (NK cells, CD4^+^ T cells, CD8^+^ T cells and B cells) did not show any signs of SARS-CoV-2 infection (**Figures 3A–C**). Fluorescence microscopy confirmed infection of monocytes and revealed that the viral N protein was localized intracellularly and partially in the endosomal compartment (**Figure 3D**).

We next determined the strength and outcome of SARS-CoV-2 infection in monocytes by infecting them with SARS-CoV-2, in parallel with the highly susceptible human colorectal carcinoma cell line Caco-2 (57). At 24 hpi, infected monocytes exhibited prominent N protein staining (**Figure 3E**), and detectable, but limited presence of dsRNA (**Figure 3F**), a hallmark of SARS-CoV-2 genome replication, that was observed in only ~10% of the cells (data not shown). In contrast, Caco-2 cells showed uniform intracellular N protein staining localized around the nucleus (**Figure 3G**) and massive staining for dsRNA, indicative of intense virus replication (**Figure 3H**). The quantification of infectious virus particles in cell culture supernatants via median tissue culture infectious dose (TCID_50_) estimation further revealed that monocytes did not give rise to progeny virus. Although low amounts of SARS-CoV-2 were detected in supernatants of infected monocytes at 24 hpi (which may reflect residual input virus), no infectious virus was quantified at 72 hpi (**Figure 3I**). Contrary to that, supernatants of infected Caco-2 cells contained substantial amounts of SARS-CoV-2 at both 24 and 72 hpi (**Figure 3I**). Altogether, these data indicate that human blood monocytes can be infected by SARS-CoV-2, but the infection is abortive.

### 3.4 SARS-CoV-2 activates monocytes via endosomal ssRNA-sensing TLRs

**Figure 4:**
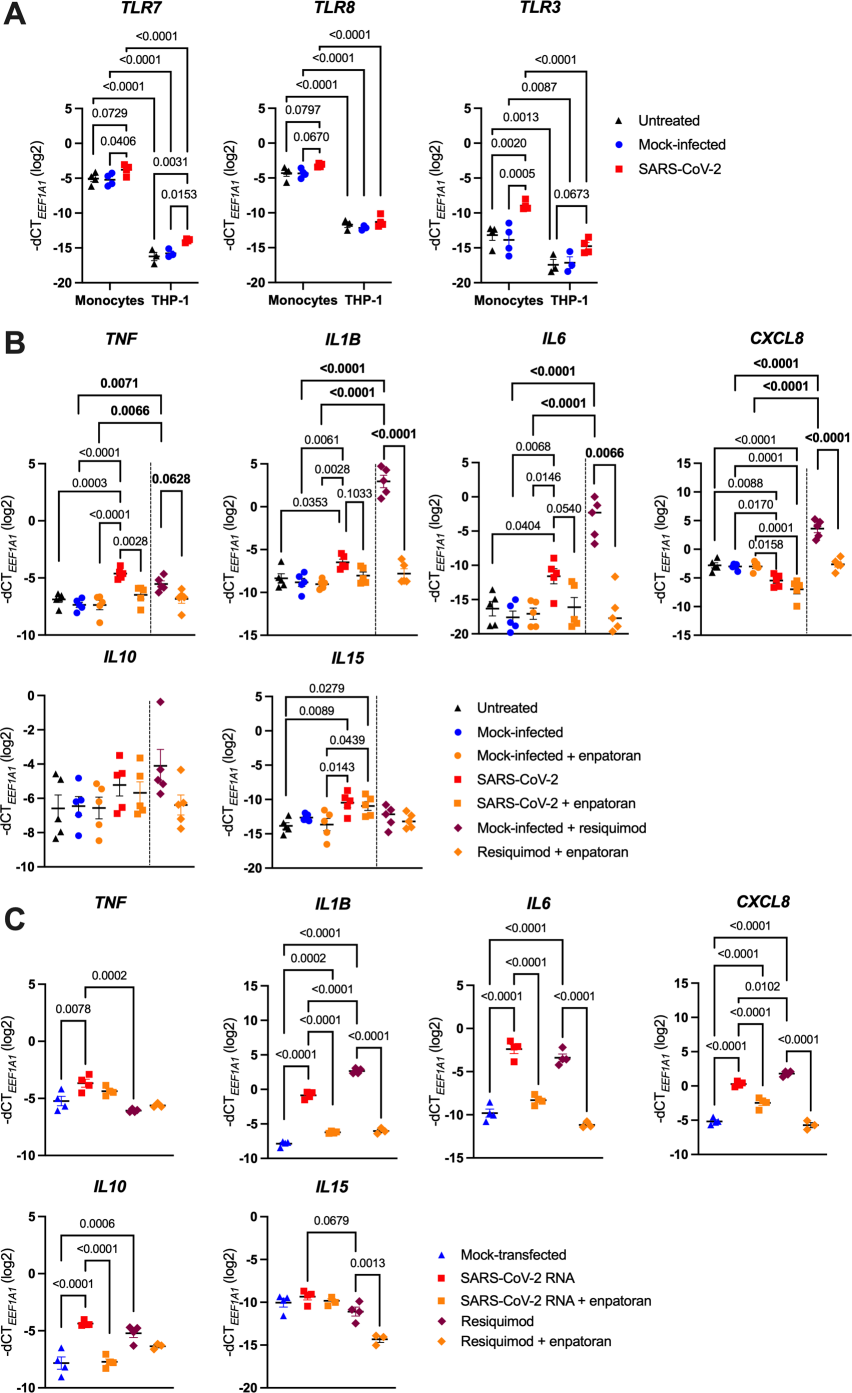
SARS-CoV-2 activates monocytes via endosomal ssRNA-sensing TLRs. **(A)** MACS-sorted human blood monocytes or THP-1 NF-κB::eGFP reporter cells were infected with SARS-CoV-2 (MOI 2), or treated with the respective mock supernatant, or left untreated, for 24 h. Expression of *TLR7*, *TLR8* and *TLR3* was measured by RT-qPCR (n = 3-4). **(B)** MACS-sorted monocytes were infected with SARS-CoV-2 at MOI 2, or the respective mock supernatant, or left untreated for 24 h, and if indicated, pretreated with the TLR7/8 antagonist enpatoran (1 μM). As controls for proper TLR7/8 stimulation, monocytes were in parallel treated with the TLR7/8 agonist resiquimod (2.5 μg/ml) for 24 h and pretreated or not with enpatoran (1 μM). *TNF, IL1B, IL6*, *CXCL8, IL10* and *IL15* mRNA levels were determined using RT-qPCR (n = 5 donors). **(C)** MACS-sorted monocytes were either mock-transfected or transfected with the *in vitro*-transcribed 4.5 kb fragment of SARS-CoV-2 RNA and were pretreated (or not) with enpatoran (1 μM). As controls, cells were also treated with resiquimod (2.5 μg/ml) in the presence or absence of enpatoran. After 24 h, *TNF, IL1B, IL6*, *CXCL8, IL10* and *IL15* mRNA levels were quantified by RT-qPCR (n = 4 donors). Data in **(A–C)** are represented as mean ± SEM and statistical significance was assessed using two-way **(A)** or one-way **(C)** ANOVA with Tukey’s *post-hoc* test, or using two independent one-way ANOVA tests with Tukey’s *post-hoc* test **(B)**, where their significances are depicted in normal or bold letters, respectively.

After infection has been established, intracellular viral RNA can be recognized by the endosomal TLR7 and 8, or TLR3, which sense ssRNA or dsRNA, respectively (22, 38). To determine whether endosomal TLRs mediate monocyte activation by SARS-CoV-2, we first verified their expression by RT-qPCR. As shown in **Figure 4A**, we detected fairly high amounts of both *TLR7* and *TLR8* transcripts, which remained unchanged upon infection. *TLR3* mRNA levels, on the other hand, were much lower and seemed induced by SARS-CoV-2, but nevertheless they were not on a par with *TLR7/8* mRNA abundance. We also profiled endosomal TLRs in the THP-1 NF-κB::eGFP reporter cells. Interestingly, *TLR7* and *TLR8* levels were significantly lower in the THP-1 cells compared to primary monocytes (**Figure 4A**), although enough to enable response to the dual TLR7/8 agonist resiquimod (R848), whereas the similarly stimulated Jurkat reporter lines were largely unresponsive (**Supplementary Figure S3**). We thus presume that these differences in *TLR7* and *TLR8* expression may contribute to the differential behavior of monocytes, THP-1 and Jurkat cells upon SARS-CoV-2 infection.

We confirmed the functionality of TLR7 and TLR8 in monocytes by treating them with resiquimod for 24 h, which led to a significant upregulation of all profiled proinflammatory cytokine genes (*TNF*, *IL1B, IL6* and *CXCL8*). In contrast, *IL10* as well as the antiviral cytokine gene *IL15* were only mildly induced (**Figure 4B**). Pretreatment of monocytes with the dual TLR7/8 inhibitor enpatoran nearly completely abrogated the resiquimod-induced changes (**Figure 4B**). Importantly, enpatoran treatment alone did not affect the baseline expression of the examined genes. To corroborate TLR7/8 involvement in SARS-CoV-2 sensing, we analyzed the cytokine response of monocytes infected with SARS-CoV-2 and of those treated with enpatoran before infection. Once again, we observed that SARS-CoV-2 significantly upregulated *TNF*, *IL1B* and *IL6*, along with the downregulation of *CXCL8*, while *IL10* levels were only slightly increased. *IL15* expression was also moderately induced by SARS-CoV-2 (**Figure 4B**). Crucially, pretreatment with enpatoran effectively mitigated SARS-CoV-2-induced changes in *TNF*, *IL1B* and *IL6* expression, though it had little effect on *CXCL8*, *IL10* and *IL15* mRNA levels (**Figure 4B**). Secondly, we generated by *in vitro* transcription a 4.5 kb SARS-CoV-2 ssRNA fragment that contained multiple “UGUGU” IFN induction motifs, which had been previously found to activate TLR7/8 (42, 44). We transfected monocytes with *in vitro-*transcribed RNA with or without pretreatment with enpatoran and assessed the response 24 h later. Also in this case, we observed a profound upregulation of *TNF*, *IL1B, IL6* and, unexpectedly, also *CXCL8* and *IL10* mRNA levels, which was mirrored by the treatment with resiquimod. In both instances, enpatoran pretreatment blunted monocyte responses (**Figure 4C**). Surprisingly, *IL15* levels were elevated also in mock-transfected monocytes and changed only minimally upon additional treatments, though enpatoran appeared to decrease *IL15* expression (**Figure 4C**). These results suggest a clear, yet not exclusive, role of TLR7 and/or TLR8 in SARS-CoV-2 sensing by human monocytes.

To further elucidate the role of TLR7 or TLR8 in the recognition of SARS-CoV-2 ssRNA, we generated novel TLR7 and TLR8 reporter cells by overexpressing either *TLR7* or *TLR8* ORF in the THP-1 NF-κB::eGFP reporter cell line. The resulting cell lines exhibited robust expression of the respective *TLR* mRNA (**Supplementary Figure S4A**) and their sensitivity to resiquimod was improved at least five- (TLR8 reporter cells) to ten-fold (TLR7 reporter cells) (**Supplementary Figures S4B, C**). Upon treatment of the parental and newly generated reporter cells with *in vitro*-transcribed SARS-CoV-2 RNA, both TLR7 and TLR8 THP-1 reporter cells upregulated eGFP and *TNF* mRNA to higher levels than the parental line (**Supplementary Figures S4D, E**), while *IL1B, IL6* and *CXCL8* mRNAs were the most highly expressed in the TLR8 THP-1 reporter cells (**Supplementary Figure S4E**). Enpatoran once again attenuated these changes (**Supplementary Figures S4D, E**). Interestingly, despite their enhanced responsiveness to the viral RNA, TLR7- and TLR8-overexpressing cells did not react to the infection with replication-competent SARS-CoV-2, since we measured only negligible eGFP expression by flow cytometry (**Supplementary Figure S4F**). This was well in line with a low infection rate, quantified by the N protein mRNA expression (**Supplementary Figure S4G**), which we also observed previously (**Figure 2E**). Additionally, expression of the cytokine genes remained low in all three reporter lines (**Supplementary Figure S4H**). Collectively, these data suggest that both TLR7 and TLR8 are involved in SARS-CoV-2 ssRNA recognition and that there is a certain preference in the subsequent cytokine response following either TLR7 or TLR8 triggering. However, in contrast to primary monocytes, THP-1 reporter cells, regardless of TLR7/8 overexpression, appear to be rather resistant to SARS-CoV-2 infection.

### 3.5 SARS-CoV-2 entry into monocytes is not mediated through the S protein RBD–ACE2 interaction

**Figure 5:**
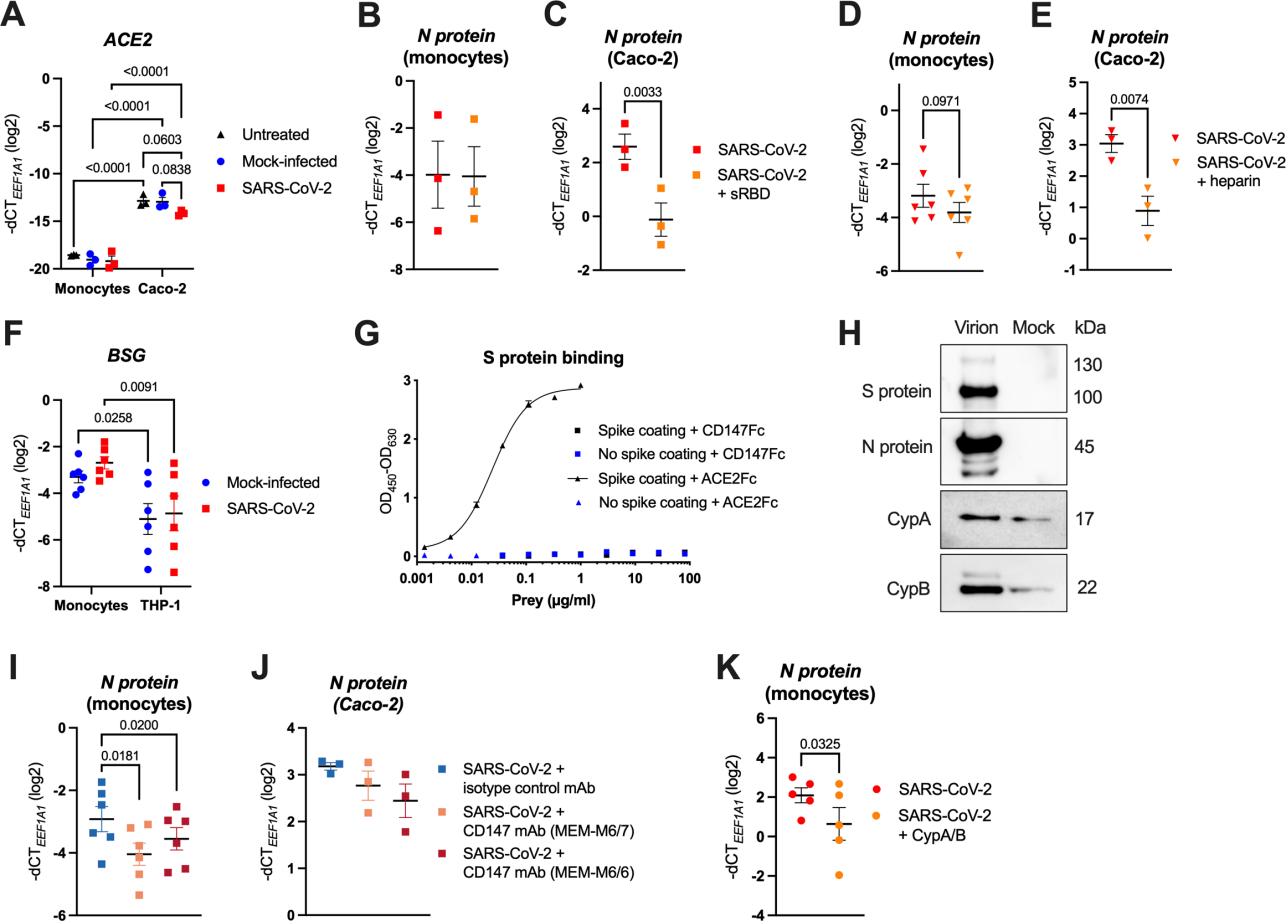
SARS-CoV-2 entry into monocytes is not mediated via the interaction of the S protein RBD with ACE2, but occurs through virion-incorporated cyclophilins binding to CD147. **(A)** MACS-sorted human blood monocytes or Caco-2 cells were infected with SARS-CoV-2 at MOI 2, or the respective mock supernatant, or left untreated, for 24 h. Expression of *ACE2* was determined by RT-qPCR (n = 3). **(B, C)** Human monocytes **(B)** and Caco-2 cells **(C)** were pretreated (or not) with sRBD (final concentration 5 μg/ml) and subsequently infected with SARS-CoV-2 at MOI 2 for monocytes and MOI 0.1 for Caco-2 cells. Excess virus was washed away after 1 h of incubation and infection was quantified by measuring SARS-CoV-2 N protein mRNA via RT-qPCR after 24 h (n = 3). **(D, E)** Human monocytes **(D)** and Caco-2 cells **(E)** were infected with SARS-CoV-2 (MOI 2 for monocytes and MOI 0.1 for Caco-2 cells) that was preincubated (or not) with heparin (final concentration 250 U/ml). Excess virus was washed away after 1 h and infection was quantified by measuring SARS-CoV-2 N protein mRNA via RT-qPCR after 24 h (n = 6 (monocytes), n = 3 (Caco-2 cells)). **(F)** Human monocytes or THP-1 cells were either mock-infected or infected with SARS-CoV-2 at MOI 2, for 24 h. Expression of *BSG* (encoding for CD147) was determined using RT-qPCR (n = 6). **(G)** His-tagged SARS-CoV-2 S protein (1 μg/ml) was coated (or not) onto Pierce Nickel-coated plates, and incubated with Fc-tagged ACE2, or Fc-tagged CD147 at the indicated concentrations. Binding was visualized by ELISA, using HRP-conjugated mouse anti-human IgG Fc mAb. **(H)** Western blot analysis of purified SARS-CoV-2 virions or mock-infected supernatant (mock) of Caco-2 cells, where SARS-CoV-2 structural proteins (S and N protein) as well as CypA and CypB were detected using specific Abs. One representative experiment of three is shown. **(I, J)** Monocytes **(I)** and Caco-2 cells **(J)** were pretreated with blocking CD147 mAbs (clones MEM-M6/7 or MEM-M6/6) or with an isotype control mAb (each at final concentration 20 μg/ml) and subsequently infected with SARS-CoV-2 at MOI 2 for monocytes and MOI 0.1 for Caco-2 cells. Excess virus was washed away after 1 h and infection was quantified by measuring SARS-CoV-2 N protein mRNA via RT-qPCR after 24 h (n = 6 (monocytes), n = 3 (Caco-2 cells)). **(K)** MACS-sorted monocytes were pretreated (or not) with recombinant cyclophilins A and B (CypA/B; each at final concentration 10 μg/ml) and infected with SARS-CoV-2 at MOI 2, and infection was quantified by measuring SARS-CoV-2 N protein mRNA via RT-qPCR after 24 h (n = 5 donors). Data in **(A–F)** and **(I–K)** are visualized as mean ± SEM, and statistical significance was assessed using two-way ANOVA with Tukey’s *post-hoc* test **(A, F)**, or paired t-test **(B–E, K)** or RM-one-way ANOVA with Dunnett’s *post-hoc* test **(I, J)**.

The above data show that in order to engage the endosomal TLR7/8, SARS-CoV-2 needs to effectively enter monocytes. However, since monocytes do not express *ACE2*, neither at the baseline, nor upon SARS-CoV-2 infection (**Figure 5A**), this entry is likely not mediated via the canonical S protein-ACE2 interaction. This is in sharp contrast to the highly SARS-CoV-2-susceptible Caco-2 cells, where we observed moderate *ACE2* mRNA levels, which showed a tendency to be downregulated upon infection (**Figure 5A**).

ACE2-independent, but S protein RBD-dependent SARS-CoV-2 infection via various alternative receptors has been described (12). To determine, whether SARS-CoV-2 entry into monocytes depends on the engagement of the RBD, we infected them as well as Caco-2 cells with SARS-CoV-2 in the presence of soluble RBD (sRBD), followed by quantification of the intracellular SARS-CoV-2 N protein RNA by RT-qPCR at 24 hpi. Importantly, sRBD did not influence the virus uptake by monocytes (**Figure 5B**), whereas infection of Caco-2 cells was decreased ~6-fold (**Figure 5C**). Moreover, heparin, which inhibits SARS-CoV-2 by binding to the viral RBD, thereby disrupting virus attachment to cell surface HSPGs and ACE2 (5–7), did not significantly alter the infection of monocytes (**Figure 5D**), contrary to Caco-2 cells (**Figure 5E**). Collectively, our results indicate that SARS-CoV-2 uses alternative, ACE2- and S protein RBD-independent, mechanisms to enter monocytes.

### 3.6 CD147 serves as an alternative SARS-CoV-2 receptor by binding to virion-incorporated cyclophilins

To explore ACE2-independent SARS-CoV-2 entry mechanisms, we first characterized the expression of postulated alternative receptors (8–10, 13, 15–20) in monocytes using RT-qPCR. Among these candidates, only *BSG* (coding for CD147), *CLEC10A* (CD301), *HSPA5* (GRP78) and *TMEM106B* were detected at high levels (**Supplementary Figure S5A**). *AXL* (14) was not tested since we previously showed that monocytes stained negative for this receptor (65). Since both GRP78 and TMEM106B are mainly located in intracellular compartments (66, 67), and exhibited similar or even higher expression in non-susceptible T cells (**Supplementary Figure S5B**; **Figures 3A–C**), it is unlikely that they serve as alternative receptors for SARS-CoV-2 in monocytes. We therefore restricted our further analyses to *BSG* (CD147) and *CLEC10A* (CD301) that we found more robustly expressed in monocytes than in T cells (**Supplementary Figure S5B**), and in case of *BSG*, also more robustly expressed in monocytes than in THP-1 cells (**Figure 5F**). However, pretreatment with a CD301 mAb failed to block virus entry into monocytes (**Supplementary Figure S6**), thereby excluding CD301 as a possible alternative receptor for SARS-CoV-2 infection.

In contrast, CD147 has been proposed as a SARS-CoV-2 receptor since the early months of the COVID-19 pandemic (13, 68), and treatment with the humanized CD147 mAb meplazumab was shown to accelerate the recovery of hospitalized COVID-19 patients in a small Phase II clinical trial (69). Others, however, disputed the direct interaction of the SARS-CoV-2 S protein with CD147 (70). Thus, we first assessed whether the two proteins interact by ELISA with a trimeric His-tagged SARS-CoV-2 S protein as bait. While we did not detect a specific interaction with the Fc-tagged CD147, Fc-tagged ACE2 used as a positive control yielded a strong binding with the EC_50_ = 24.23 ng/ml (**Figure 5G**). Nonetheless, CD147 may still facilitate SARS-CoV-2 infection, but through an alternative mechanism, which was described for the phylogenetically related SARS-CoV. In that, CD147 mediated virus uptake by recognizing its well-known interaction partner cyclophilin A (CypA) (71, 72) that was incorporated into SARS-CoV virions (73). To test whether also SARS-CoV-2 particles contain CypA, or the other well-known CD147 binding partner cyclophilin B (CypB) (71, 72), we isolated infectious virions and mock supernatant produced by Caco-2 cells, using a previously described serial purification approach (62, 63). As expected, SARS-CoV-2 S and N proteins were exclusively detected in the isolated virions, determined by Western blotting (**Figure 5H**). Importantly, although also detectable in the mock samples due to their known association with extracellular vesicles (74), cyclophilins were notably enriched in the isolated virions (**Figure 5H**). Furthermore, both cyclophilins and the related CypD, which has not yet been reported to interact with CD147, were significantly enriched in the proteome of SARS-CoV-2 virions purified from supernatants of infected Caco-2 cells, as determined by mass spectrometry. However, neither CD147 nor ACE2 were enriched in virion preparations (**Supplementary Table S2**) (62). These data corroborate that SARS-CoV-2 incorporates CypA, CypB and the lesser known CypD into its virions.

To test whether CD147 mediates SARS-CoV-2 uptake into monocytes by recognizing its binding partners CypA and CypB incorporated in SARS-CoV-2 virions, we pretreated isolated monocytes with two mAbs targeting the CD147 IgII domain (MEM-M6/6 and MEM-M6/7), or with an isotype control mAb, followed by infection with SARS-CoV-2. Based on the mapped interaction sites of cyclophilins with CD147 that reside in and adjacent to the IgII domain (71, 72, 75), the employed CD147 mAbs should effectively block their interaction. By quantification of the SARS-CoV-2 N protein RNA in infected monocytes at 24 hpi, we observed that both CD147 mAbs significantly decreased SARS-CoV-2 uptake (on average, ~1.6 to 2.2-fold) in comparison to the treatment with the isotype control mAb (**Figure 5I**). Interestingly, the CD147 blocking mAbs also mildly, but not significantly, reduced the infection of Caco-2 cells (**Figure 5J**). Finally, we disrupted the interaction between monocyte-expressed CD147 and virion-incorporated cyclophilins by pretreating monocytes with recombinant human CypA and CypB and subsequently infected them with SARS-CoV-2. Recombinant cyclophilins used as a decoy likewise significantly decreased the SARS-CoV-2 uptake (on average, ~2.7-fold), as determined by the quantification of SARS-CoV-2 N protein RNA 24 hpi (**Figure 5K**). In summary, these data indicate that CD147, through the interaction with virion-incorporated cyclophilins, serves as an alternative SARS-CoV-2 receptor on human monocytes.

## 4 Discussion

SARS-CoV-2 infection and the resulting epithelial damage triggers migration of circulating monocytes and other circulating immune cells to the affected tissues, guided by gradients of proinflammatory cytokines, and thereby increasing the numbers of the defending mononuclear cells and enhancing inflammation (1, 23). Activation of these innate immune cells in turn promotes the development of adaptive T cell and B cell responses that are necessary for virus clearance (1).

Here we show that human blood monocytes can effectively sense SARS-CoV-2, and subsequently respond by substantial upregulation of the proinflammatory cytokines TNF, IL-1β and IL-6, which comprise the core hyperinflammatory signature seen in severely ill COVID-19 patients (1, 45, 76). We observed that SARS-CoV-2 sensing is not mediated via engagement of the well-known and highly expressed pattern recognition receptors TLR1, TLR2, TLR4 and TLR6 (64). These cell surface-expressed TLRs have been proposed as the main sensors of either the SARS-CoV-2 S, N or E protein by others (28–35, 37). In our hands, however, neither recombinant trimeric SARS-CoV-2 S protein nor SARS-CoV-2 N protein profoundly activated human monocytes and the monocytic THP-1 NF-κB::eGFP reporter cell line (**Figure 1**). Similarly, the highly sensitive Jurkat NF-κB::eGFP reporter cell lines also reacted relatively poorly to these recombinant proteins, leading to less than three-fold increase in the eGFP signal (**Figure 1**), and more importantly, they failed to activate when stimulated with replication-competent SARS-CoV-2 (**Figure 2C**). Since our Jurkat reporter cells are able to sense their prototype ligands in the range of pg/ml (55), we presume that the weak response to the recombinant SARS-CoV-2 proteins might stem from negligible endotoxin contamination (for example, the S protein used in our studies was declared by the manufacturer to contain <0.1 EU/1 µg protein, which is in line with our own measurements of 0.021 EU/1 µg protein, using Limulus amebocyte lysate assay). Our data are thus well in accordance with the work of Cinquegrani et al., who treated human monocyte-derived macrophages with recombinant SARS-CoV-2 S proteins from multiple commercial sources, scrutinizing their endotoxin levels, and observing that macrophage activation in fact correlated with endotoxin contamination of the recombinant proteins, or with the lack of glycosylation of *E. coli*-produced S proteins (77). Nevertheless, the situation might be more complex *in vivo* since the S protein has been shown to bind LPS with high affinity and to boost proinflammatory responses of myeloid cells to low LPS levels via the TLR4-NF-κB axis (78). These interactions might also help explain the excessive inflammation observed in COVID-19 patients with compromised gut barrier integrity (79, 80).

Most importantly, we have discovered that monocytes mount a proinflammatory response to SARS-CoV-2 following virus uptake via an alternative mechanism involving CD147 and virus-hijacked host cellular cyclophilins (**Figures 2D, E**; **3**; **5**) and stimulation of the endosomal ssRNA-sensing TLR7 and TLR8 (**Figure 4**). Stimulation of both primary monocytes and THP-1 reporter cell lines with SARS-CoV-2-derived ssRNA led to profound upregulation of all measured cytokine genes (**Figure 4C**; **Supplementary Figure S4E**), while primary monocytes increased *TNF*, *IL1B* and *IL6* and decreased *CXCL8* expression upon infection with replication-competent SARS-CoV-2 (**Figures 2D**; **4B**). This dichotomous response might perhaps be explained by the action of microRNAs miR-17 and miR-93-5p, which inhibit CXCL8 expression (81) and were found to be significantly increased upon SARS-CoV-2 infection (82, 83). Whether this is indeed the case, remains to be addressed in future studies.

SARS-CoV-2 RNA has been consistently detected in lung monocytes, macrophages and dendritic cells of COVID-19 patients using single-cell RNA sequencing (46–48). Myeloid cells are considered to be devoid of the prime SARS-CoV-2 receptor ACE2 (16, 18, 20, 36, 46–48, 51), and initially, *in vitro* infection experiments provided contrasting results whether and how these cells are infected. At low MOIs, usually around 0.01, the direct infection of monocytes, monocyte-derived macrophages and dendritic cells, or even primary alveolar macrophages with replication-competent SARS-CoV-2 was not observed, unless myeloid cells were cocultured with infected epithelial cells that enabled indirect infection through phagocytosis (36, 50, 51). In contrast, monocytes, monocyte-derived macrophages or pDCs were successfully infected with SARS-CoV-2 at MOI 0.1-1, or more (9, 16, 19, 37, 47, 49, 84, 85). This suggests that SARS-CoV-2 infection of myeloid cells via alternative mechanisms is less efficient than the infection via ACE2, or the infection is more difficult to track, since it is not productive (18, 49, 84, 85). We, too, observed that human monocytes could be efficiently, yet abortively, infected with SARS-CoV-2 at MOI 2, as we detected high levels of both the N protein RNA and protein intracellularly, and even observed dsRNA intermediates indicative of SARS-CoV-2 replication, but no increasing titers of infectious virus over time (**Figure 3**). The high doses of SARS-CoV-2, which are required for the monocyte infection are in line with the alternative, i.e., ACE2- and S protein-independent entry mechanism mediated via the interaction of virus-associated cyclophilins and monocyte-expressed CD147, since even the combined amount of CypA and CypB present in SARS-CoV-2 virions is naturally lower than the amount of the S protein. Cyclophilins are evolutionary conserved, ubiquitously expressed proteins with peptidyl-prolyl *cis-trans* isomerase (PPIase) activity that function as chaperones and assist conformational folding of not only cellular but also viral proteins (71, 72, 86). They were discovered as specific ligands for the immunosuppressive drug cyclosporin A, which inhibits their PPIase activity and consequently viral replication within cells (86). Due to interaction with viral structural proteins, CypA and CypB have been shown to be integrated into virions of several enveloped viruses, such as SARS-CoV, HIV-1, vaccinia virus or measles virus (73, 86–88), and allowed virus infection by binding to their cognate receptor CD147. Incorporation of CypA versus CypB seems to be virus-specific: CypA-CD147 interaction enables SARS-CoV and HIV-1 infection (73, 87), while CypB-CD147 interaction has been shown to facilitate infection of measles virus (88), and in both cases, virus infectivity is hindered by cyclosporin A treatment (87, 88). Naturally, this alternative infection mechanism has also been tested for SARS-CoV-2 (89). Even though the usage of a CD147 blocking mAb at 1 µg/ml to inhibit the CypA-CD147 interaction was not effective at preventing SARS-CoV-2 infection of the ACE2-positive Calu-3 lung cell line, a highly effective *BSG* knockdown using a specific siRNA was (89). The authors concluded that CD147 downregulation affected viral infection indirectly and not via the CypA-CD147 interaction, since they also observed lower ACE2 levels upon *BSG* knockdown and both receptors were considerably decreased upon SARS-CoV-2 infection (89). Instead, we observed that two CD147 blocking mAbs at a concentration of 20 µg/ml mildly hindered SARS-CoV-2 infection of ACE2^+^ Caco-2 cells, while they significantly reduced infection of ACE2^-^ human monocytes. Similarly, recombinant cyclophilins curtailed monocyte infection. These findings suggest that CD147 serves as an alternative SARS-CoV-2 receptor by recognizing virus-hijacked cyclophilins in ACE2^-^ cells, whereas this mechanism plays only a minor role in cells that are positive for the prime SARS-CoV-2 receptor, ACE2. Nevertheless, neither CD147 blocking mAbs at 20 µg/ml nor recombinant CypA and CypB, each at 10 µg/ml, completely prevented monocyte infection by SARS-CoV-2, suggesting that either higher concentrations are needed, or other alternative receptors are involved.

CD147 is a broadly expressed member of the immunoglobulin superfamily of receptors that is involved in many physiological processes, such as lymphocyte responsiveness, fertilization, neurological functions at early stages of development, angiogenesis, cell adhesion, invasion and matrix metalloproteinase (MMP) secretion, apoptosis as well as regulation of energy metabolism due to its association with monocarboxylate transporters and amino acid transporters. Because of its multifaceted roles and the ability to trigger a proinflammatory response, overexpressed CD147 was associated with several diseases, including atherosclerosis, ischemic myocardial injury, heart failure, rheumatoid arthritis, or cancer, some of which are associated with higher risk for severe COVID-19 (71, 72, 90). For example, CD147 signaling in monocytes and macrophages upon extracellular CypA binding causes inflammation by inducing migration and activation of MMPs, leading to atherosclerotic foam cell and plaque formation (91). Treatments with a CD147 mAb or the small molecule SP-8356 disrupting the CypA-CD147 interaction have been shown to suppress atherosclerosis (92). Moreover, CypA binding to CD147 led to reactive oxygen species production and metabolic dysfunction in T cells from COVID-19 patients, which could be mitigated by cyclosporin A treatment (93). The effectiveness of cyclosporin A in lowering serum hyperinflammation-associated cytokines and chemokines was later demonstrated in a small cohort of hospitalized patients with COVID-19 (94). Similarly, the CD147 mAb meplazumab was found to inhibit SARS-CoV-2 infection *in vitro*, though the authors pinpointed the effects to blocking SARS-CoV-2 S protein binding to CD147 (13). Subsequently, meplazumab was clinically tested in hospitalized COVID-19 patients and proved effective in reducing viral loads and severity of the disease with a good safety, tolerance, and pharmacokinetic profile (69). The same positive results have been reported in the follow-up multicenter phase 2/3 randomized double-blind clinical trial with severe COVID-19 patients, which additionally revealed reduced mortality and cytokine levels upon meplazumab administration (95).

Collectively, based on our data and the above-mentioned studies we conclude that SARS-CoV-2 infection of human monocytes, and possibly also of other myeloid cells via hijacked cyclophilins that effectively bind to myeloid-expressed CD147, contributes to hyperinflammation observed in COVID-19 patients, and that targeting the cyclophilin-CD147 axis might therefore represent another approach for alleviating the severe disease course of COVID-19. Further studies are needed to identify susceptible patients for this core hyperinflammatory response, e.g., using a recently developed skin prick test to determine signaling downstream of PRRs, including endosomal TLRs (96). Identifying such vulnerable patients prone to a hyperinflammatory sentinel myeloid immune cell signature or alternatively, profoundly impaired response, as seen in patients with TLR7 loss-of-function variants (97, 98), may allow clinicians to refer to additional prophylactic or therapeutic approaches.

## Data Availability

We reused a previously published mass spectrometry dataset (62) of the proteome of SARS-CoV-2 virions or the respective mock supernatant deposited in the ProteomeXchange Consortium (http://www.ebi.ac.uk/pride/archive/) under the identifier PXD050009.
