# Supplementary material for: Cyclophilin–CD147 interaction enables SARS-CoV-2 infection of human monocytes and their activation via Toll-like receptors 7 and 8
Source: Front Immunol. 2025 Feb 3;16:1460089. doi: 10.3389/fimmu.2025.1460089 (PMC11830813; doi:10.3389/fimmu.2025.1460089)
Supplement: Supplementary file 1 [file DataSheet1.pdf]

Supplementary Material

Cyclophilin–CD147 interaction enables SARS-CoV-2 infection of human monocytes and their activation via Toll-like receptors 7 and 8

Gabor Tajti[#], Laura Gebetsberger[#], Gregor Pamlitschka, Katharina Aigner-Radakovics, Judith Leitner, Peter Steinberger, Hannes Stockinger and Anna Ohradanova-Repic[*]

[#] Contributed equally

[*]Correspondence: anna.repic@meduniwien.ac.at

1 Supplementary Figures and Tables

Table S1. Detailed list of reagents used in the study

Reagent type	Designation	Source or reference	Catalog number	Additional information
Antibody	PE-Cy7-conjugated CD3 mouse mAb (clone UCHT1)	BioLegend	300420	
Antibody	Brilliant Violet 750-conjugated CD4 mouse mAb (clone SK3)	BioLegend	344644	
Antibody	PerCP-conjugated CD8 mouse mAb (clone SK1)	BioLegend	344708	
Antibody	Brilliant Violet 421-conjugated CD14 mouse mAb (clone M5E2)	BioLegend	301830	
Antibody	PerCP-Cy5.5-conjugated CD16 mouse mAb (clone 3G8)	BioLegend	302028	
Antibody	Brilliant Violet 605-conjugated CD19 mouse mAb (clone HIB19)	BioLegend	302244	
Antibody	PE-conjugated CD56 mouse mAb (clone 5.1H11)	BioLegend	362508	
Antibody	Alexa Fluor 647-conjugated anti-human TLR2 mAb (clone TL2.1)	BioLegend	309714	
Antibody	Alexa Fluor 488-conjugated anti-LAMP-1 mouse mAb (clone H4A3)	BioLegend	328610	
Antibody	Brilliant Ultraviolet 805-conjugated CD14 mouse mAb (clone M5E2)	BD Biosciences	612902	
Antibody	Anti-SARS-CoV-2 N protein rabbit mAb (clone #019)	SinoBiological	40143-R019	
Antibody	Anti-SARS-CoV-2 N protein recombinant chimeric mAb (clone mBG17)	Absolute Antibody	Ab02382-23.0	Mouse IgG1 Fab, rabbit IgG1 Fc
Antibody	Anti-dsRNA mouse mAb (clone J2)	Jena Bioscience	RNT-SCI-10010200	
Antibody	Alexa Fluor 647-conjugated goat anti-mouse IgG (H+L) secondary Ab	Invitrogen	A-21236	
Antibody	Alexa Fluor 647-conjugated goat anti-rabbit IgG (H+L) secondary Ab	Invitrogen	A-21245	
Antibody	HRP-conjugated mouse anti-human IgG Fc mAb (clone 50B4A9)	GeneScript	A01854	
Antibody	Anti-Cyclophilin A rabbit polyclonal Ab (C1C3)	GeneTex	GTX104698	

Antibody	Anti-Cyclophilin B rabbit polyclonal Ab	Invitrogen	PA1-027A
Antibody	Anti-SARS-CoV-2 S protein mouse mAb (clone #42)	SinoBiological	40591-MM42
Antibody	Anti-SARS-CoV-2 N protein mouse mAb (clone #05)	SinoBiological	40143-MM05
Antibody	Functional grade anti-human/mouse TLR2 recombinant human Ab, REAfinity (clone REA109)	Miltenyi Biotec	130-098-855
Antibody	REA control recombinant human Ab, REAfinity (clone REA293)	Miltenyi Biotec	130-129-977
Antibody	CD147 mouse anti-human mAb (clone MEM-M6/6)	Kind gift from Prof. V. Horejsi, Institute of Molecular Genetics of the Czech Academy of Sciences, Prague, Czechia	N.A.
Antibody	CD147 mouse anti-human mAb (clone MEM-M6/7)	Kind gift from Prof. V. Horejsi	N.A.
Antibody	CD8 mouse anti-human mAb (clone MEM-87)	Kind gift from Prof. V. Horejsi	N.A.
Antibody	CD16 mouse anti-human mAb (clone MEM-154)	Kind gift from Prof. V. Horejsi	N.A.
Antibody	CD19 mouse anti-human mAb (clone WIN-19)	Kind gift from Prof. V. Horejsi	N.A.
Antibody	CD20 mouse anti-human mAb (clone MEM-97)	Kind gift from Prof. V. Horejsi	N.A.
Antibody	CD56 mouse anti-human mAb (clone MEM-188)	Kind gift from Prof. V. Horejsi	N.A.
Antibody	Isotype control mouse mAb (clone PPV06)	Kind gift from Prof. V. Horejsi	N.A.
Antibody	CD301 mouse anti-human mAb (H037G3)	BioLegend	354702
Antibody	Ultra-LEAF™ Purified Mouse IgG2a, κ Isotype Ctrl Antibody	BioLegend	400264
Antibody	Alkaline phosphatase-conjugated goat anti-rabbit IgG (whole molecule) secondary Ab	Sigma-Aldrich	A3687
Antibody	Peroxidase-conjugated rabbit anti-Mouse IgG (whole molecule) secondary Ab	Sigma-Aldrich	A9044
Dye	LIVE/DEAD™ Fixable Blue Dead Cell Stain Kit	Invitrogen	L23105
Dye	4′,6-diamidino-2-phenylindole (DAPI; for DNA staining)	Sigma-Aldrich	D5942
Commercial assay or kit	Total RNA Purification Mini Spin Kit	Genaxxon	S5304

Commercial assay or kit	RNA Isolation, Total RNA Kit, peqGOLD	VWR	13-6834-02	
Commercial assay or kit	M-MuLV Reverse Transcriptase	New England Biolabs	M0253	
Commercial assay or kit	High-Capacity cDNA Reverse Transcription Kit	Applied Biosystems	4368814	
Commercial assay or kit	Luna® Universal qPCR Master Mix	New England Biolabs	M3003	
Commercial assay or kit	Luna® Universal Probe qPCR Master Mix	New England Biolabs	M3004	
Commercial assay or kit	CD14 MicroBeads human	Miltenyi Biotec	130-050-201	
Commercial assay or kit	Anti-Mouse IgG MicroBeads	Miltenyi Biotec	130-048-401	
Agonist/ antagonist	Pam3CSK4	InvivoGen	tlrl-pms	
Agonist/ antagonist	FSL-1	InvivoGen	tlrl-fsl	
Agonist/ antagonist	LPS Ultrapure	InvivoGen	tlrl-3pelps	
Agonist/ antagonist	Resiquimod (R848)	InvivoGen	tlrl-r848	
Agonist /antagonist	Enpatoran hydrochloride (M5049)	MedChemExpress	HY-134581A	
Protein	Recombinant SARS-CoV-2 S Protein RBD (carrier-free) (sRBD)	BioLegend	793606	
Protein	CD147Fc	Acro Biosystems	CD7-H5259	
Protein	ACE-2 Fc Chimera, Human	GenScript	Z03516	
Protein	Recombinant SARS-CoV-2 S protein, S1+S2 (R683A, R685A) Trimer (CF)	BioLegend	795806	
Protein	SARS-CoV-2 Nucleocapsid (aa1-419), His Tag Recombinant Protein	Invitrogen	RP-87707	
Protein	Recombinant human macrophage colony-stimulating factor (M-CSF)	Novo-Nordisk		
Protein	Recombinant Cyclophilin A/PPIA Protein, Human	MedChemExpress	HY-P70047	
Protein	Recombinant Cyclophilin B/PPIB Protein, Human, His-tagged	MedChemExpress	HY-P70011A	
Sequence-based reagent	TNFf	This study, synthesized by Sigma-Aldrich	N.A.	GCTGCACTTTGGAGTGATCG
Sequence-based reagent	TNFr	This study, synthesized by Sigma-Aldrich	N.A.	TCAGCTTGAGGGTTTGCTACA

Sequence-based reagent	IL1Bf	This study, synthesized by Sigma-Aldrich	N.A.	AGCTCGCCAGTGAAATGATG
Sequence-based reagent	IL1Br	This study, synthesized by Sigma-Aldrich	N.A.	GTGGTGGTCGGAGATTCGTAG
Sequence-based reagent	CXCL8f	This study, synthesized by Sigma-Aldrich	N.A.	TGGCAGCCTTCCTGATTT
Sequence-based reagent	CXCL8r	This study, synthesized by Sigma-Aldrich	N.A.	TTTGGGGTGGAAAGGTTTG
Sequence-based reagent	IL10f	Ref. 52	N.A.	TCCCTGTGAAAACAAGAGCAAG
Sequence-based reagent	IL10r	Ref. 52	N.A.	AGTCGCCACCCTGATGTCTC
Sequence-based reagent	IL6f	This study, synthesized by Sigma-Aldrich	N.A.	TAGTGAGGAACAAGCCAGAGC
Sequence-based reagent	IL6r	This study, synthesized by Sigma-Aldrich	N.A.	TTGGGTCAGGGGTGGTTATT
Sequence-based reagent	IL15f	This study, synthesized by Sigma-Aldrich	N.A.	ACAGAAGCCAACTGGGTGAA
Sequence-based reagent	IL15r	This study, synthesized by Sigma-Aldrich	N.A.	CATCTCCGGACTCAAGTGAAAT
Sequence-based reagent	EEF1A1f	Ref. 52	N.A.	GTGCTAACATGCCTTGGTTC
Sequence-based reagent	EEF1A1r	Ref. 52	N.A.	AGAACACCAGTCTCCACTCG
Sequence-based reagent	ACE2f	This study, synthesized by Sigma-Aldrich	N.A.	GCAGCCACACCTAAGCATTT
Sequence-based reagent	ACE2r	This study, synthesized by Sigma-Aldrich	N.A.	GAGTGCTTGTTTGAGCAGGA
Sequence-based reagent	BSGf	This study, synthesized by Sigma-Aldrich	N.A.	GACGACCAGTGGGGAGAGTA
Sequence-based reagent	BSGr	This study, synthesized by Sigma-Aldrich	N.A.	CGTTGATGTGTTCTGACGACTTC
Sequence-based reagent	AGERf	This study, synthesized by Sigma-Aldrich	N.A.	TCAGGACCAGGGAACCTACA
Sequence-based reagent	AGERr	This study, synthesized by Sigma-Aldrich	N.A.	CAGGGCCAGGGCTAGAGT
Sequence-based reagent	ASGR1f	This study, synthesized by Sigma-Aldrich	N.A.	GGAGGCAATGTGGGAAGAA
Sequence-based reagent	ASGR1r	This study, synthesized by Sigma-Aldrich	N.A.	CAGACACGAACTGCTTCACG
Sequence-based reagent	CD209f	This study, synthesized by Sigma-Aldrich	N.A.	CTTCACCTGGATGGGACTTTC
Sequence-based reagent	CD209r	This study, synthesized by Sigma-Aldrich	N.A.	GGGCTCTCCTCTGTTCCAATA
Sequence-based reagent	CLEC10Af	This study, synthesized by Sigma-Aldrich	N.A.	GCTGGTCATCATCTGTGTGGT

Sequence-based reagent	CLEC10Ar	This study, synthesized by Sigma-Aldrich	N.A.	GATCTCCGCCACAGTGTTTG
Sequence-based reagent	CLEC4Gf	This study, synthesized by Sigma-Aldrich	N.A.	GTTACTGGCTGGGCCTGAG
Sequence-based reagent	CLEC4Gr	This study, synthesized by Sigma-Aldrich	N.A.	AGCGTCATTGGGCTCTCC
Sequence-based reagent	CLEC4Mf	This study, synthesized by Sigma-Aldrich	N.A.	AGCAGCAGCAAATCTATCAAGAAC
Sequence-based reagent	CLEC4Mr	This study, synthesized by Sigma-Aldrich	N.A.	AGTTCCGCTGGGAGTTAGACA
Sequence-based reagent	HSPA5f	This study, synthesized by Sigma-Aldrich	N.A.	AACCGCTGAGGCTTATTTGG
Sequence-based reagent	HSPA5r	This study, synthesized by Sigma-Aldrich	N.A.	AGTTCCAGCGTCTTTGGTTG
Sequence-based reagent	KREMEN1f	This study, synthesized by Sigma-Aldrich	N.A.	TCAGACTGTCCCAGGTAGCAAT
Sequence-based reagent	KREMEN1r	This study, synthesized by Sigma-Aldrich	N.A.	GGATGAGGAGAGTTGCCAGAC
Sequence-based reagent	NRP1f	This study, synthesized by Sigma-Aldrich	N.A.	ATCCTCATCGGGCATTCTCT
Sequence-based reagent	NRP1r	This study, synthesized by Sigma-Aldrich	N.A.	TGCCCAGAGCTTCCATACAT
Sequence-based reagent	TMEM106Bf	This study, synthesized by Sigma-Aldrich	N.A.	TCGACGTGAAATACATTGGTGT
Sequence-based reagent	TMEM106Br	This study, synthesized by Sigma-Aldrich	N.A.	TTGAACTTGGGCAGTGATGTT
Sequence-based reagent	SARS-COV-2 Nf	Ref. 61, synthesized by Sigma-Aldrich	N.A.	GACCCCAAAATCAGCGAAAT
Sequence-based reagent	SARS-COV-2 Nr	Ref. 61, synthesized by Sigma-Aldrich	N.A.	TCTGGTTACTGCCAGTTGAATCTG
Sequence-based reagent	SARS-COV-2 N probe	Ref. 61, synthesized by Sigma-Aldrich	N.A.	FAM-ACCCCGCATTACGTTTGGTGGACC-BHQ1
Sequence-based reagent	TLR3f	This study, synthesized by Sigma-Aldrich	N.A.	GCGCTAAAAAGTGAAGAACTGG
Sequence-based reagent	TLR3r	This study, synthesized by Sigma-Aldrich	N.A.	CGTGAAAACACCCTGGAGAA
Sequence-based reagent	TLR7f	This study, synthesized by Sigma-Aldrich	N.A.	CTTCAACCAGACCTCTACATTCC
Sequence-based reagent	TLR7r	This study, synthesized by Sigma-Aldrich	N.A.	AAACCATCTAGCCCCAAGGA
Sequence-based reagent	TLR8f	This study, synthesized by Sigma-Aldrich	N.A.	GACCCAACTTCGATACCTAAACC
Sequence-based reagent	TLR8r	This study, synthesized by Sigma-Aldrich	N.A.	ATGCCCCAGAGGCTATTTCT

Sequence-based reagent	flTLR7f	This study, synthesized by Sigma-Aldrich	N.A.	ACCAGACCTCTACATTCCATTTTG
Sequence-based reagent	flTLR7r	This study, synthesized by Sigma-Aldrich	N.A.	AGGGCTAGACCGTTTCCTTG
Sequence-based reagent	flTLR8f	This study, synthesized by Sigma-Aldrich	N.A.	TTGAAAGGGAGAATGAAGGAGTC
Sequence-based reagent	flTLR8r	This study, synthesized by Sigma-Aldrich	N.A.	TCATTCCTTTGCATCTTTATTATGG
Sequence-based reagent	NSP4-9f	This study, synthesized by Sigma-Aldrich	N.A.	**CAACATG**GGTGGTAAAATTGTTAATAATTGGTTG
Sequence-based reagent	NSP4-9r	This study, synthesized by Sigma-Aldrich	N.A.	**TTA**TTGTAGACGTACTGTGGCAGCTA
Sequence-based reagent	Random heptamers (7N)	Synthesized by Sigma-Aldrich	N.A.	
Reagent	Heparin sodium salt from porcine intestinal mucosa	Sigma-Aldrich	H3149	
Reagent	ECL™ Prime Western Blotting Detection Reagent	Cytiva	RPN2236	
Reagent	Substrate Reagent Pack for ELISA	R&D Systems	DY999	
Reagent	Penicillin-Streptomycin (10,000 U/mL)	Gibco/Thermo Fisher Scientific	15140122	
Reagent	HEPES solution 1 M, pH 7.0-7.6	Sigma-Aldrich	H0887	
Reagent	MEM non-essential amino acids solution (100X)	Gibco/Thermo Fisher Scientific	11140035	
Reagent	Dimethyl sulfoxide (DMSO), cell culture grade	PanReac AppliChem	A3672	
Reagent	Lymphoprep™	Axis-Shield	1858	
Reagent	L-Glutamine (200 mM)	Gibco/Thermo Fisher Scientific	25030081	
Reagent	Fetal Bovine Serum (FBS) South America, Heat Inactivated	Biowest	S181H	
Reagent	Deoxynucleotide (dNTP) Solution Mix	New England Biolabs	N0447	
Reagent	RNase Inhibitor, Human Placenta	New England Biolabs	M0307	
Reagent	Phusion High-Fidelity DNA Polymerase	New England Biolabs	M0530S	
Commercial assay or kit	Quick Blunting Kit	New England Biolabs	E1201	
Commercial assay or kit	HiScribe T7 Quick High Yield RNA Synthesis Kit	New England Biolabs	E2050S	
Commercial assay or kit	Monarch DNA Gel Extraction Kit	New England Biolabs	T1020S	
Commercial assay or kit	Monarch Plasmid Miniprep Kit	New England Biolabs	T1010S	

Plasmid	pBluescript II KS(-) Phagemid vector	Stratagene	212208	
Plasmid	pBMN-Z	Addgene	Plasmid #1734	A gift from Garry Nolan (Addgene plasmid # 1734; http://n2t.net/addgene:1734; RRID:Addgene_1734)
Plasmid	pMD_OGP (gag.pol retroviral packaging plasmid)	kindly provided by Dr. RC Mulligan, Harvard Medical School, Boston, MA, USA	N.A.	
Plasmid	pMD2.G (VSV-G envelope expressing plasmid)	Addgene	Plasmid #12259	A gift from Didier Trono (Addgene plasmid # 12259; http://n2t.net/addgene:12259; RRID:Addgene_12259)
Reagent	TurboFect Transfection Reagent	Thermo Fisher Scientific	R0531	
Reagent	Lipofectamine 3000 Transfection Reagent	Thermo Fisher Scientific	L3000001	
Reagent	DEPC-treated nuclease-free water	Fischer Scientific	BP561-1	
Reagent	Beriglobin® P (human immunoglobulin)	CSL Behring	N.A.	
Reagent	CC/Mount™	Sigma-Aldrich	C9368	
Other	DMEM, high glucose, GlutaMAX™ supplement, pyruvate	Gibco/Thermo Fisher Scientific	31966021	
Other	RPMI 1640 medium	Gibco/Thermo Fisher Scientific	21875034	

Table S2. Presence of cyclophilin A, B and D (encoded by *PPIA*, *PPIB* or *PPID*, respectively), CD147 (two isoforms, produced by alternative promoter usage from *BSG*), ACE2 or SARS-CoV-2 S and N proteins in the proteomic analysis of purified SARS-CoV-2 virions or the respective mock supernatant of the Caco-2 producer cells. Shown data are extracted from the mass spectrometry analysis of L. Gebetsberger et al., 2024 (Ref. 62), the raw data of which were deposited in the ProteomeXchange Consortium via the PRIDE partner repository (http://www.ebi.ac.uk/pride/archive/) under the identifier PXD050009. n.d. not detected.

Accession (Uniprot)	Gene	Relative abundance in the virus preparation (± SEM)	Relative abundance in the mock preparation (± SEM)	Abundance in the virus fraction (log2 fold-change)	p value
**P62937-2**	***PPIA***	128.963 (± 12.29)	71.037 (± 5.96)	0.860	0.037
**P23284**	***PPIB***	132.889 (± 12.28)	67.111 (± 2.50)	0.986	0.039
**Q08752**	***PPID***	139.593 (± 11.90)	60.407 (± 6.46)	1.208	0.014
**P35613**	***BSG***	102.594 (± 18.80)	97.406 (± 12.57)	0.075	0.854
**P35613-3**	***BSG***	106.153 (± 12.42)	93.847 (± 11.10)	0.178	0.558
**Q9BYF1**	***ACE2***	n. d.	n. d.		
**P0DTC9**	**N**	199.907 (± 11.28)	0.093 (± 0.01)	11.075	0.004
**P0DTC2**	**S**	199.717 (± 14.49)	0.283 (± 0.01)	9.465	0.007

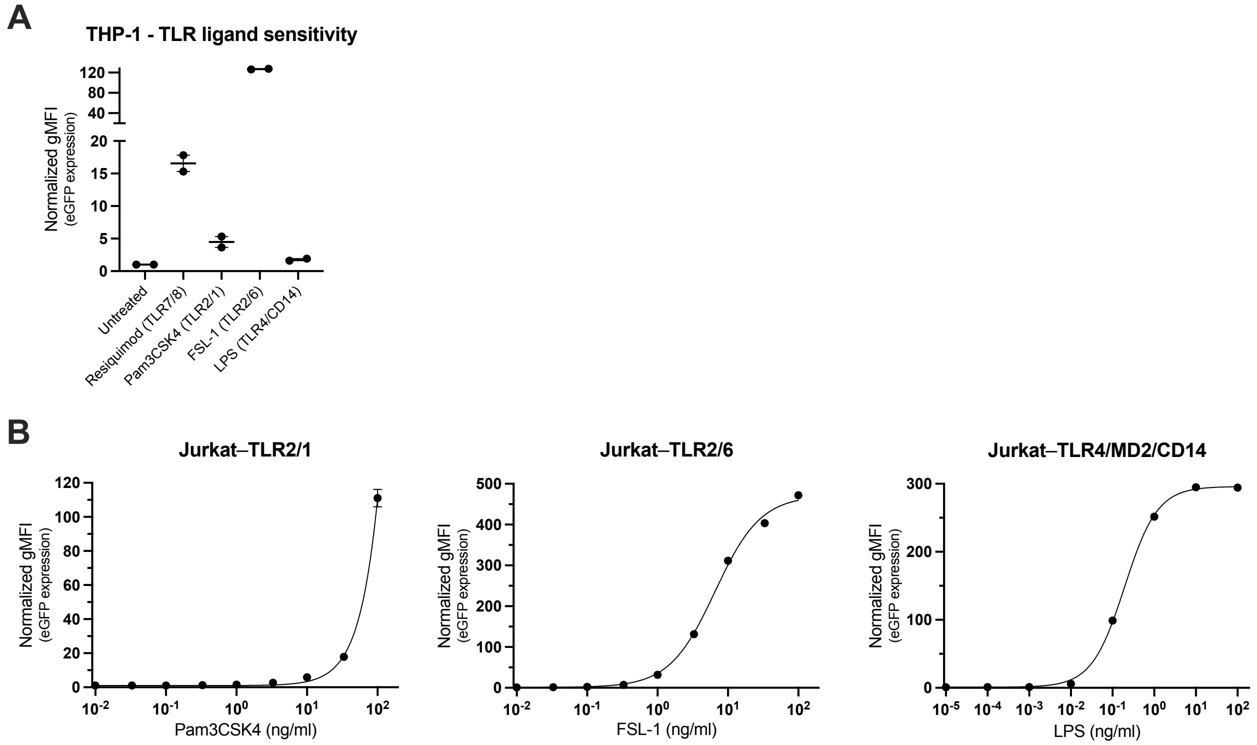

Figure S1. Stimulation of NF-κB::eGFP reporter cell lines with the prototype TLR ligands shows higher sensitivity of the Jurkat reporter cells to the designated ligands than the broadly-reacting THP-1 cells. (A) THP-1 NF-κB::eGFP reporter cells were left untreated or stimulated with the TLR7/8 ligand resiquimod (2.5 µg/ml), TLR2/1 ligand Pam3CSK4 (10 ng/ml), TLR2/1 ligand FSL-1 (10 ng/ml) or TLR4 ligand LPS (10 ng/ml). **(B)** Jurkat NF-κB::eGFP reporter cells engineered to express the indicated set of TLRs were treated with the prototype ligands at the indicated concentrations. In each case, GFP expression was measured by flow cytometry after 24 h, and shown as fold-change of geometric mean of fluorescence intensity (gMFI). Data are shown as a scatter plot and mean ± SEM from 2 individual experiments (A), or as dose-response curves generated with 4-parameter sigmoidal fit with mean ± SEM (B, n = 2).

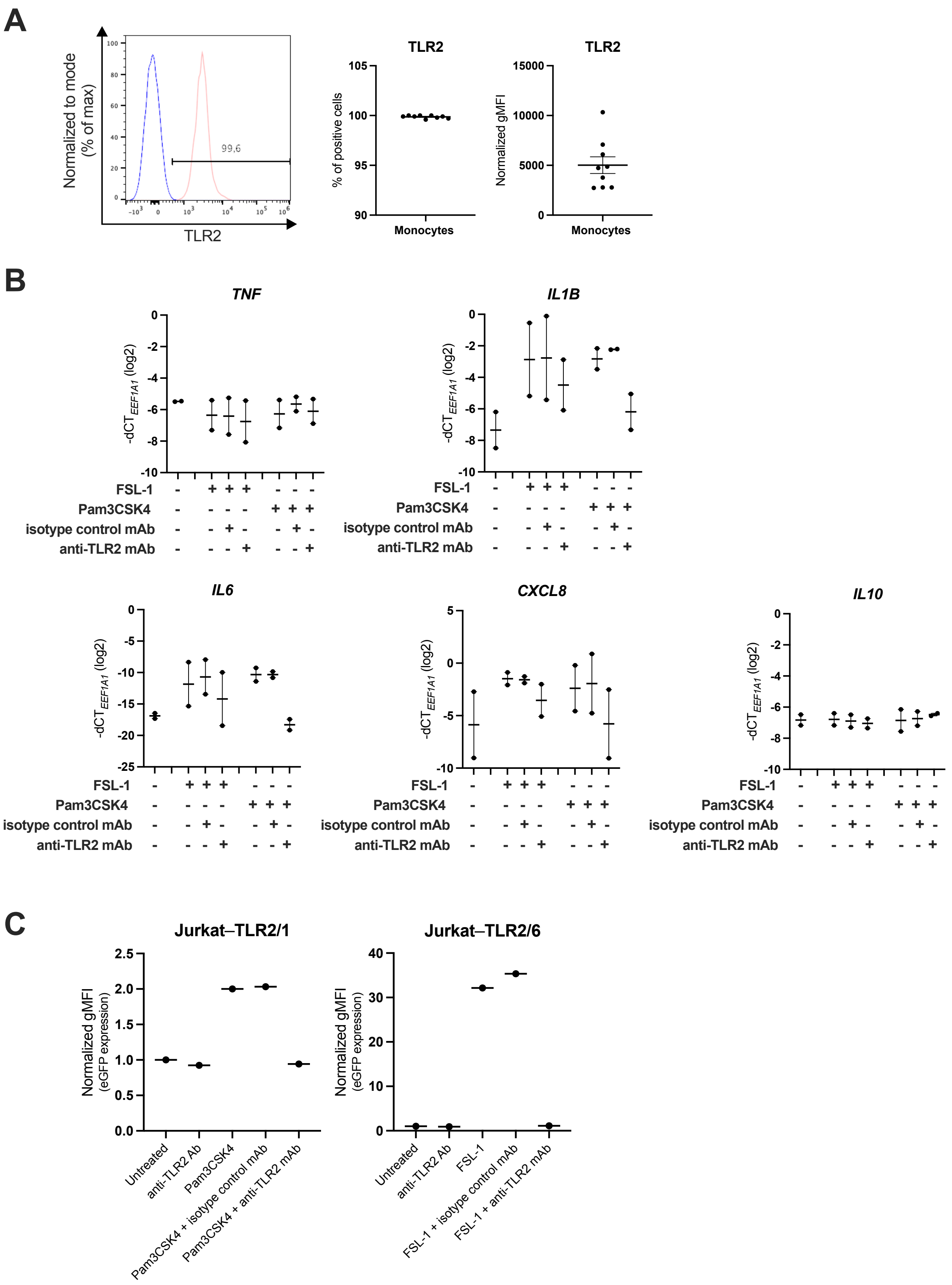

Figure S2. The anti-TLR2 blocking antibody is functional, since it counteracts the effects of the TLR2/1 and TLR2/6 ligands in primary monocytes and Jurkat NF-κB::eGFP reporter cells. (A)

TLR2 expression in primary human monocytes, gated as $CD3^-CD14^+$ in PBMC samples was determined by flow cytometry. A representative experiment and pooled data of 9 donors, evaluated as percentage of $TLR2^+$ monocytes and normalized gMFI are shown as scatter plots with individual points and mean ± SEM. **(B)** Human peripheral blood monocytes were stimulated with Pam3CSK4 (10 ng/ml, TLR2/1 agonist) or FSL-1 (10 ng/ml, TLR2/6 agonist) in the presence or absence of anti-TLR2 mAb or isotype control mAb (10 µg/ml) for 24 h, and expression of the indicated proinflammatory cytokine genes was measured by RT-qPCR. Gene expression is relative to the *EEF1A1* housekeeping gene and is shown as a scatter plot with individual values (n = 2 donors) and mean ± SEM. Statistical analysis was not performed. **(C)** Jurkat NF-*κ*B::eGFP reporter cells overexpressing either TLR2/1 or TLR2/6 were either left untreated or treated for 24 h with the prototype ligands, the TLR2/1 agonist Pam3CSK4 (2 ng/ml), or the TLR2/6 agonist FSL-1 (2 ng/ml). In parallel, cells were pretreated with either anti-TLR2 mAb or isotype control mAb (both at 10 µg/ml) for 30 min before addition of the agonists. eGFP expression was measured by flow cytometry after 24 h and is shown as fold-change of gMFI. Data are visualized as a scatter plot from 1 experiment.

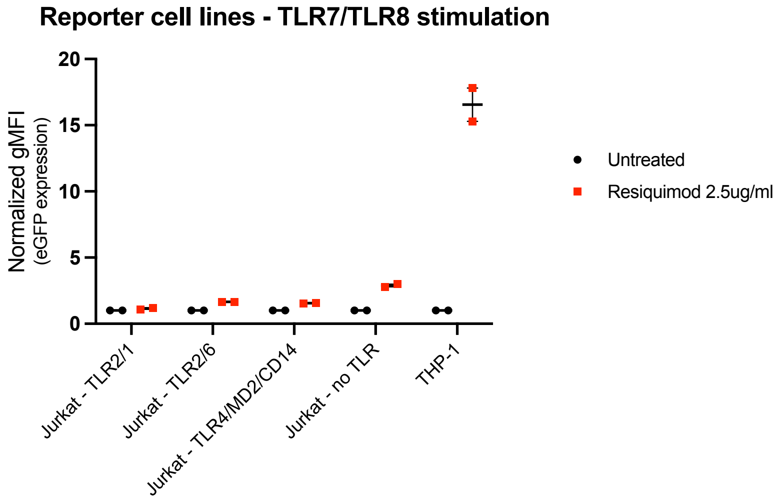

Figure S3. THP-1 but not the Jurkat NF-*κ*B::eGFP reporter cells respond to the TLR7/8 stimulation. Jurkat NF-*κ*B::eGFP reporter cells overexpressing the indicated surface TLRs (or not) as well as THP-1 NF-*κ*B::eGFP reporter cells were treated (or not) with the TLR7/8 agonist resiquimod (2.5 µg/ml) for 24 h. eGFP expression was measured by flow cytometry, and reported as fold-change of gMFI to untreated cells in scatter plots with individual values and mean ± SEM (n = 2).

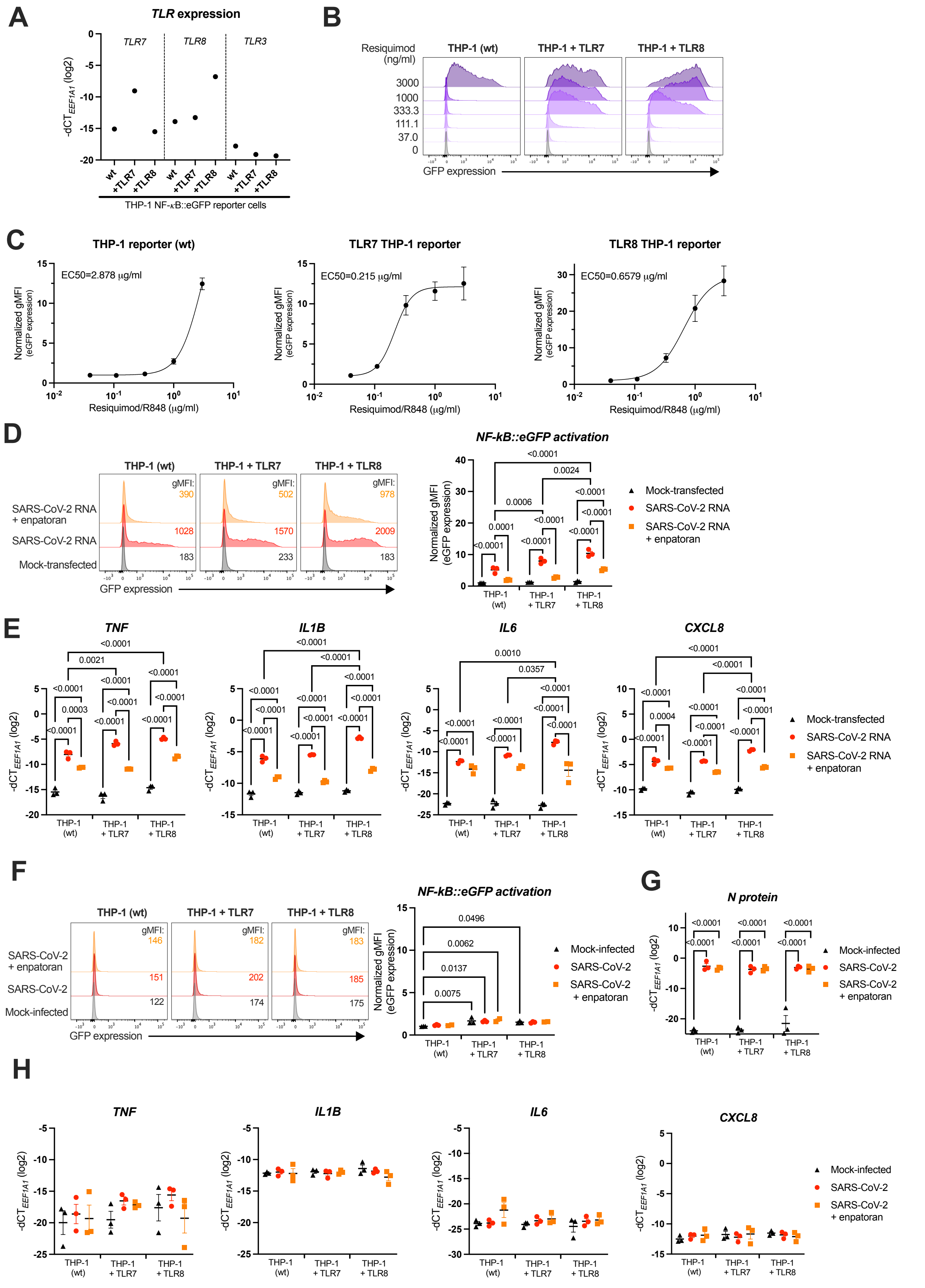
A
TLR expression
TLR7
TLR8
TLR3
-dCT EEF1A1 (log2)
wt
+TLR7
+TLR8
THP-1 NF-κB::eGFP reporter cells
B
Resiquimod (ng/ml)
THP-1 (wt)
THP-1 + TLR7
THP-1 + TLR8
3000
1000
333.3
111.1
37.0
0
GFP expression
C
THP-1 reporter (wt)
EC50=2.878 μg/ml
TLR7 THP-1 reporter
EC50=0.215 μg/ml
TLR8 THP-1 reporter
EC50=0.6579 μg/ml
Normalized gMFI (eGFP expression)
Resiquimod/R848 (μg/ml)
D
SARS-CoV-2 RNA + enpatoran
SARS-CoV-2 RNA
Mock-transfected
gMFI: 390
1028
183
gMFI: 502
1570
233
gMFI: 978
2009
183
NF-kB::eGFP activation
Mock-transfected
SARS-CoV-2 RNA
SARS-CoV-2 RNA + enpatoran
E
TNF
IL1B
IL6
CXCL8
THP-1 (wt)
THP-1 + TLR7
THP-1 + TLR8
F
SARS-CoV-2 + enpatoran
SARS-CoV-2
Mock-infected
gMFI: 146
151
122
gMFI: 182
202
174
gMFI: 183
185
175
Mock-infected
G
N protein
H

Figure S4. Generation, characterization and stimulation of the novel TLR7 and TLR8 THP-1 NF-*κ*B::eGFP reporter cells with SARS-CoV-2 *in vitro*-transcribed ssRNA or live SARS-CoV-2. **(A)** Novel TLR7 and TLR8 THP-1 NF-*κ*B::eGFP reporter cells were prepared by retroviral transduction of the respective ORFs, followed by sorting of resiquimod-responsive cells. Expression of *TLR7*, *TLR8* and *TLR3* relative to the *EEF1A1* housekeeping gene in wild-type (wt) or TLR7 or TLR8-overexpressing THP-1 NF-*κ*B::eGFP reporter cells was subsequently measured by RT-qPCR (n = 1). **(B)** Wild-type (wt) or TLR7- or TRL8-overexpressing THP-1 NF-*κ*B::eGFP reporter cells were treated with resiquimod at the indicated concentrations. eGFP expression was measured by flow cytometry after 24 h and representative histograms from one of three independent experiments are shown. **(C)** eGFP expression from (B), expressed as fold-change of gMFI was used to construct dose-response curves generated with 4-parameter sigmoidal fit with mean ± SEM and calculate half maximal effective concentration (EC50; n = 3). **(D-E)** THP-1 reporter cells were either mock-transfected, or transfected with the 4.5 kb fragment of *in vitro*-transcribed SARS-CoV-2 RNA, and a parallel sample was pretreated with enpatoran (1 μM) before SARS-CoV-2 RNA delivery. 24 h later, eGFP expression was measured by flow cytometry (D) and expression of *TNF, IL1B, IL6* and *CXCL8* mRNA levels were determined using RT-qPCR (E). In (D), representative histograms (left) from one experiment and pooled data (right) from three independent experiments are shown. **(F)** THP-1 reporter cells were either mock-infected, or infected with SARS-CoV-2 (MOI = 2-3), and a parallel sample was pretreated with enpatoran (1 μM) before SARS-CoV-2 infection. After 24 h, cells were fixed and eGFP expression was measured by flow cytometry. Representative histograms (left) from one experiment and pooled data (right) from three independent experiments are shown. **(G-H)** THP-1 reporter cells were infected as described in (F) and total RNA was isolated 24 hpi. Infection was quantified by measuring SARS-CoV-2 N protein mRNA via RT-qPCR (G) and cell response was determined based on expression of *TNF, IL1B, IL6* and *CXCL8* mRNA (H), also via RT-qPCR (n = 3 independent experiments). Data in (D-H) are shown as scatter plots with individual values and mean ± SEM, and statistical significance was assessed using two-way ANOVA with Tukey's *post-hoc* test.

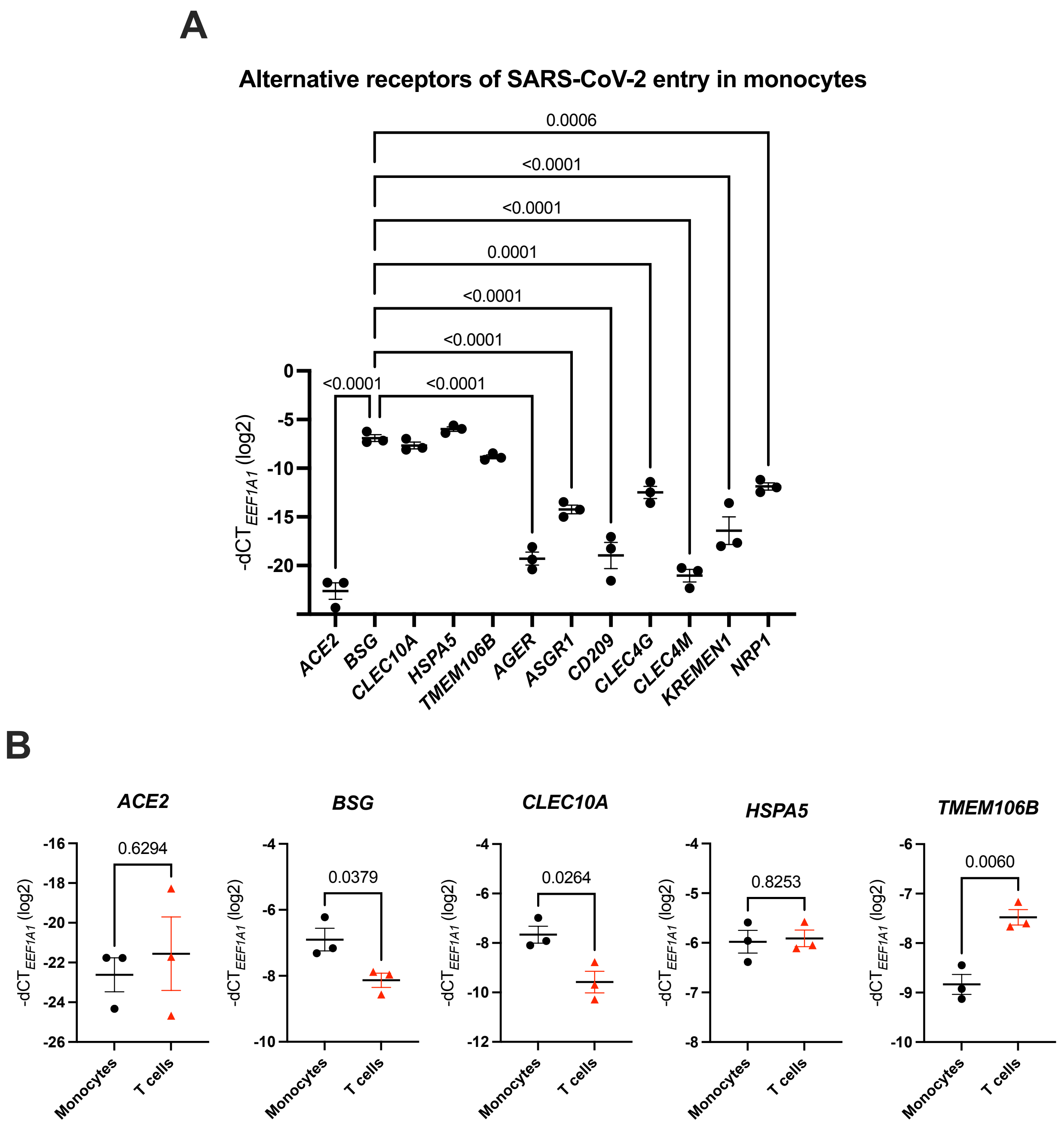

Figure S5. Expression of *ACE2* and alternative SARS-CoV-2 entry receptors in monocytes and CD4$^+$ T cells. (A) Expression of *ACE2* and other proposed alternative SARS-CoV-2 entry receptors relative to the *EEF1A1* housekeeping gene in MACS-sorted human peripheral blood monocytes was determined by RT-qPCR (n = 3). **(B)** Expression of *ACE2* and the genes encoding highly abundant alternative receptors (*BSG*, *CLEC10A*, *HSPA5* and *TMEM106B*) relative to *EEF1A1* was determined in human peripheral blood monocytes and in autologous CD4$^+$ T cells by RT-qPCR (n = 3). Data in (A) and (B) are shown as scatter plots with individual values and mean ± SEM. In (A), expression of all determined genes was compared to *BSG* expression using one-way ANOVA with Dunnett's *post-hoc* test. In (B), gene expression between monocytes and T cells was statistically evaluated using unpaired t-test (B).

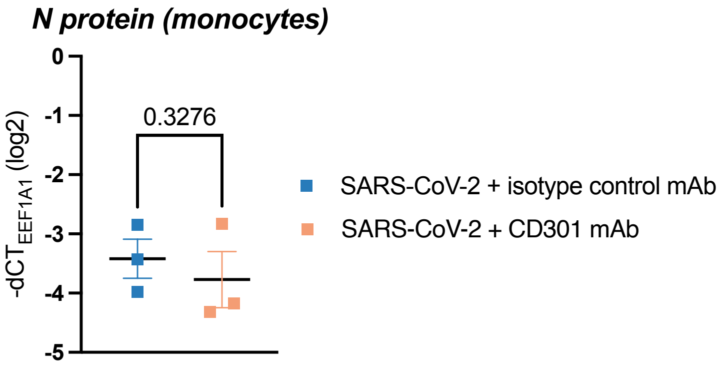

Figure S6. The CD301 antibody does not block SARS-CoV-2 entry to monocytes. MACS-sorted human peripheral blood monocytes were pretreated with the CD301 mAb H037G3 or with an isotype control mAb (each at final 20 µg/ml) and subsequently infected with SARS-CoV-2 at MOI 2. After 24 h, infection was determined by quantification of SARS-CoV-2 N protein mRNA relative to *EEF1A1* mRNA by RT-qPCR (n = 3). Results are shown as a scatter plot with individual values and mean ± SEM. Data were compared with paired t-test.
